# Octacarbonyl Ion Complexes of Actinides [An(CO)_8_]^+/−^ (An=Th, U) and the Role of f Orbitals in Metal–Ligand Bonding

**DOI:** 10.1002/chem.201902625

**Published:** 2019-08-23

**Authors:** Chaoxian Chi, Sudip Pan, Jiaye Jin, Luyan Meng, Mingbiao Luo, Lili Zhao, Mingfei Zhou, Gernot Frenking

**Affiliations:** ^1^ School of Chemistry, Biological and Materials Sciences Jiangxi Key Laboratory for Mass Spectrometry and Instrumentation East China University of Technology Nanchang Jiangxi Province 330013 China; ^2^ Institute of Advanced Synthesis School of Chemistry and Molecular, Engineering Jiangsu National Synergetic Innovation Center for, Advanced Materials Nanjing Tech University Nanjing 211816 China; ^3^ Department of Chemistry Collaborative Innovation Center of Chemistry for Energy Materials Shanghai Key Laboratory of Molecular Catalysis and Innovative Materials, Fudan University Shanghai 200433 China; ^4^ Fachbereich Chemie Philipps-Universität Marburg Hans-Meerwein-Strasse 4 35043 Marburg Germany

**Keywords:** actinides, bonding analysis, electronic structure, IR spectroscopy, octacarbonyl complexes

## Abstract

The octacarbonyl cation and anion complexes of actinide metals [An(CO)_8_]^+/−^ (An=Th, U) are prepared in the gas phase and are studied by mass‐selected infrared photodissociation spectroscopy. Both the octacarbonyl cations and anions have been characterized to be saturated coordinated complexes. Quantum chemical calculations by using density functional theory show that the [Th(CO)_8_]^+^ and [Th(CO)_8_]^−^ complexes have a distorted octahedral (*D*_4*h*_) equilibrium geometry and a doublet electronic ground state. Both the [U(CO)_8_]^+^ cation and the [U(CO)_8_]^−^ anion exhibit cubic structures (*O_h_*) with a ^6^A_1g_ ground state for the cation and a ^4^A_1g_ ground state for the anion. The neutral species [Th(CO)_8_] (*O_h_*; ^1^A_1g_) and [U(CO)_8_] (*D*_4*h*_; ^5^B_1u_) have also been calculated. Analysis of their electronic structures with the help on an energy decomposition method reveals that, along with the dominating 6d valence orbitals, there are significant 5f orbital participation in both the [An]←CO σ donation and [An]→CO π back donation interactions in the cations and anions, for which the electronic reference state of An has both occupied and vacant 5f AOs. The trend of the valence orbital contribution to the metal–CO bonds has the order of 6d≫5f>7s≈7p, with the 5f orbitals of uranium being more important than the 5f orbitals of thorium.

## Introduction

The classification of chemical elements in the periodic table, introduced 150 years ago by Mendeleyev,^1^ is based on the electronic shell structure of the atoms, which is a decisive factor for their chemical bonds. This very useful concept divides the chemical elements into main‐group atoms with a (*n*)s(*n*)p valence shell, transition metals with a (*n*)s(*n*)p(*n*−1)d valence shell, and the lanthanides and actinides with a (*n*)s(*n*)p(*n*−1)d(*n*−2)f valence shell. The associated electron‐counting rules, specifically, octet rule for main‐group atoms, 18‐electron rule for transition metals, and 32‐electron rule for lanthanides and actinides, had already been suggested by Langmuir in 1921 prior to the advent of modern quantum chemistry.^2^

The relative contribution of the different valence orbitals towards bonding interaction is an important factor to determine the chemical behavior of the atoms. The (*n*)s and (*n*)p atomic orbitals (AOs) of the main‐group atoms are both very important for the chemical bonds, with the first octal‐row elements exhibiting a special role owing to the similar radii of the s and p functions.^3^ The (*n*)p AOs of the heavier main‐group atoms with *n*>2 are significantly larger than the respective (*n*)s AOs, which leads to significantly different bonds and increasing stability of the lower oxidation states of the heavier atoms; this is further enhanced by the filling of the d shells and relativistic effects for the very heavy atoms.^4^ For transition metals, the (*n*−1)d valence orbitals are usually much more important than the (*n*)s and (*n*)p AOs.^5^ It has even been suggested that (*n*)p AOs are not genuine valence orbitals for transition metals and that the 18‐electron rule should be replaced by a 12‐electron rule based on a (*n*)s(*n*−1)d valence shell.^6^ This has been rejected by several authors in favor of the original 18‐electron rule.^7^ The great relevance of the (*n*)p AOs of the transition metals has recently been demonstrated.^8^

The relevance and the role of the f orbitals for the chemical bonds in lanthanide and actinide compounds is less clear. Pseudopotentials, in which the f electrons are part of the effective nuclear potential, have a similar accuracy as those with explicit consideration of f electrons in the valence shell.^9^ This could be taken as evidence that f orbitals are not genuine valence orbitals for the 4f‐shell elements. In a recent analysis of octacarbonyl anion complexes of the late lanthanides [Ln(CO)_8_]^−^ (Ln=Tm, Yb, Lu), we found that the 32‐electron rule is valid in the systems that have 32–34 valence electrons when the symmetry of the orbitals is considered.^10^ The energy contribution of the f electrons and the acceptor strength of the vacant f orbitals were found to be very small. The analysis of the electronic structures revealed that the metal–CO bonding interactions in the [Ln(CO)_8_]^−^ anion complexes are dominated by [Ln(d)]→(CO)_8_ π backdonation and [Ln(d)]←(CO)_8_ σ donation. The metal f orbitals play a very minor role in the interatomic interactions, because the 4f orbitals of the lanthanides have a strongly contracted radial distribution.

We extended our work to carbonyl complexes of the early actinide atoms thorium and uranium. Previously, stable heteroleptic uranium carbonyl complexes, such as [(C_5_Me_5_)_3_U(CO)], [(C_5_H_4_SiMe_3_)_3_U(CO)], and [(C_5_Me_4_H)_3_U(CO)], have been isolated,^11^ and homoleptic thorium and uranium carbonyl complexes An(CO)_*n*_ (*n*=1–6) have been produced in solid noble gas matrices.^12^ A saturated coordinated [U(CO)_8_]^+^ cation complex has also been studied in the gas phase by using infrared photodissociation spectroscopy.^13^ These uranium carbonyl complexes exhibit redshifted carbonyl stretching frequencies that are comparable to those seen for neutral transition‐metal carbonyl ligands with significant backbonding. A molecular thorium carbonyl compound [(C_5_Me_5_)_3_Th(CO)][BPh_4_], which is isolable at room temperature, has also been reported and reveals a slightly redshifted carbonyl absorption at 2131 cm^−1^.^14^ Theoretical calculations suggest that back‐donation from f orbitals is quite efficient in the uranium carbonyl complexes, even though the spatial overlap is rather different from that of d orbitals.11c, 15 In contrast, the back‐donation only arises from (C_5_Me_5_)_3_‐based orbitals in the thorium carbonyl complex.^14^

Herein, we report an infrared photodissociation spectroscopic and theoretical study of actinide octacarbonyl ion complexes [An(CO)_8_]^*q*^ (An=Th, U, *q*=±1) in the gas phase. The molecules formally possess 19–23 electrons in the metal valence shell, which is far from an ideal 32 electron configuration. We report the spectroscopic identification of the complexes [An(CO)_8_]^*q*^, and we analyze the nature of the metal–CO bonds with the f orbital participation in the metal–CO bonds being quantitatively explored. For completion, we also report the computed results of the neutral octacarbonyl complexes [Th(CO)_8_] and [U(CO)_8_]. We address the question about the validity of the electron‐counting rules for the actinide octacarbonyl ions. Recent studies by us showed that the 18‐electron rule for transition‐metal complexes is valid for the formal 20‐electron systems [TM(CO)_8_]^−^ (TM=Sc, Y, La) when the symmetry of the valence orbitals is considered.^16^ The latter finding contributed to the observation of the alkaline earth octacarbonyl complexes [M(CO)_8_] (M=Ca, Sr, Ba), which have cubic (*O_h_*) symmetry and a triplet (X^3^A_1g_) electronic ground state.^17^

## Experimental and Computational Methods

The infrared photodissociation spectra of the actinide metal carbonyl complexes were measured by a collinear tandem time‐of‐flight mass spectrometer, which has been described in detail previously.^18^ The charged complexes were prepared by pulsed laser ablation of a rotating metal target by using a 1064 nm laser (fundamental of a Continuum Minilite II Nd:YAG laser) with an energy of 15–20 mJ/pulse and a repetition rate of 10 Hz. The nascent ablated plasma was entrained by 5–10 % CO seeded in a helium carrier gas expanded from a pulsed valve (General Valve, series 9) at a backing pressure of 0.5–1.0 MPa. After free expansion, the generated ions were skimmed and mass separated by a primary time‐of‐flight mass spectrometer (TOFMS). The ions of interest were then mass‐selected with a mass gate and decelerated into the extraction region of a second collinear TOFMS, in which they were irradiated by a tunable IR laser beam that was generated by a KTP/KTA/AgGaSe2 optical parametric oscillator/amplifier system (OPO/OPA, Laser Vision) pumped by a Nd:YAG laser. The wavenumber is calibrated by the photoacoustic spectrum of CO gas. Resonant absorption leads to fragmentation of the ion complex. The resulting fragment and parent ions were then reaccelerated and mass analyzed by the second TOFMS. Infrared photodissociation spectra were obtained by monitoring the yield of the fragment ions as a function of the dissociation IR laser wavenumber and normalizing to the parent ion signal. The spectra were recorded by scanning the dissociation laser in 2 cm^−1^ per step and averaged by 300–600 shots at each step.

Quantum chemical calculations that used density functional theory (DFT) were carried out to obtain the equilibrium geometries and vibrational spectra of the actinide complexes [An(CO)_8_]^+/−^ (An=Th, U), including the neutral ones. The optimizations at different spin states were performed at the B3LYP‐D3(BJ)^19^, ^20^/def2‐TZVPPD^21^/ECP level,^22^ at which the def2‐TZVPPD basis set was used for carbon and oxygen atoms, and the Stuttgart RSC 1997 ECP basis set was used for the An atom. The latter basis set uses relativistic effective small‐core (60 core electrons) potentials for the An atom. Additional energy calculations were carried out by using coupled‐cluster theory at the CCSD(T) level^23^ with the smaller def2‐SVP basis set^24^ by using the B3LYP optimized geometries. All these calculations were carried out by using the Gaussian 16 software package.^25^ A superfine integration grid was used for the calculations. For all calculations, default convergence criteria in Gaussian 16 was used, which employs RMS force criterion (ConvF) of 3×10^−4^ and RMS displacement (ConvX) of 4*ConvF as threshold values. The maximum force and the maximum displacement are scaled as 1.5*ConvF and 1.5*ConvX.

The bonding situation was studied by energy decomposition analysis (EDA)^26^ together with the natural orbitals for chemical valence (NOCV)^27^ method by using the ADF 2017.01 program package.^28^ The EDA‐NOCV^29^ calculations were performed at the B3LYP‐D3(BJ)/TZ2P^30^ level by using the B3LYP‐D3(BJ)/def2‐TZVPPD/ECP optimized geometries, in which the scalar relativistic effects were included for An by adopting the zeroth‐order regular approximation (ZORA).^31^ In the EDA method, the intrinsic interaction energy (Δ*Ε*_int_) between two fragments is decomposed into four energy components [Eq. (1)]:

(1) $$\Delta E_{int}=\Delta E_{elstat}+\Delta E_{Pauli}+\Delta E_{disp}+\Delta E_{orb}$$

The Δ*E*_elstat_ term represents the quasiclassical electrostatic interaction between the unperturbed charge distributions of the prepared fragments. The Pauli repulsion Δ*E*_Pauli_ is the energy change associated with the transformation from the superposition of the unperturbed electron densities of the isolated fragments to the wave function, which properly obeys the Pauli principle through explicit antisymmetrization and renormalization of the product wave function. The Δ*E*_disp_ term corresponds to the dispersion interaction between the fragments. The term Δ*E*_orb_ originates from the mixing of orbitals, charge transfer, and polarization between the isolated fragments.

The combination of EDA with the NOCV method allows us to partition the total Δ*E*_orb_ term into pairwise contributions of the orbital interactions. The electron density deformation Δ*ρ_k_*(*r*), which originates from the mixing of the orbital pairs *ψ_k_*(*r*) and *ψ*_−*k*_(*r*) of the interacting fragments in the complex, gives the direction and the shape of the charge flow owing to the orbital interactions [Eq. (2)], whereas the associated orbital energy term reflects the strength of such orbital interactions [Eq. (3)]. The terms *F* ^TS^_−*k*, −*k*_ and *F* ^TS^_*k*,*k*_ are diagonal transition‐state (TS) Kohn–Sham matrix elements that correspond to NOCVs with the respective eigenvalues *v_k_*.^29^

(2) $$\Delta \rho_{\mathrm{orb}}\left(r\right)=\sum_{k}\Delta \rho_{k}\left(r\right)\sum_{k=1}^{N/2}\nu_{k}\left[-\Psi_{-k}^{2}\left(r\right)+\Psi_{k}^{2}\left(r\right)\right]$$

(3) $$\Delta E_{\mathrm{orb}}=\sum_{k}\Delta E_{k}^{\mathrm{orb}}=\sum_{k=1}^{N/2}\nu_{k}\left[-F_{-k,-k}^{TS}+F_{k,k}^{TS}\right]$$

Therefore, both qualitative (Δ*ρ*_orb_) and quantitative (Δ*E*_orb_) information of the strength of individual pairs of orbital interactions can be obtained from an EDA‐NOCV analysis. For further details on the EDA‐NOCV method and its application to the analysis of the chemical bond, some recent reviews are recommended.^32^

## Results and Discussion

**Figure 1:**
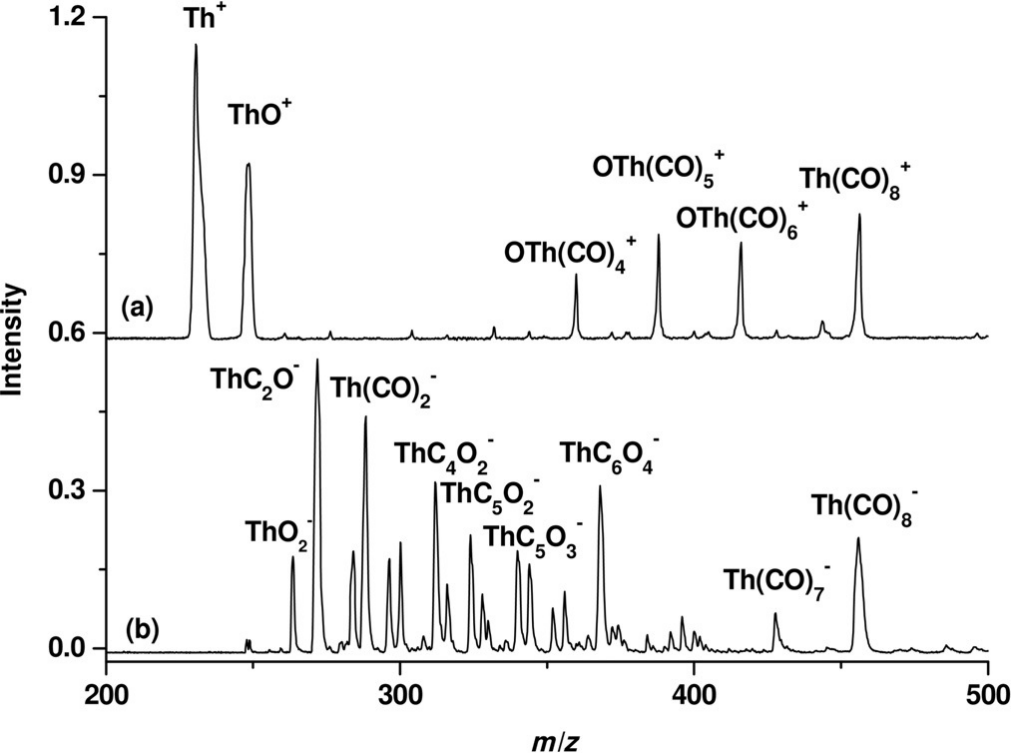
Mass spectra of a) thorium carbonyl cation and b) anion complexes.

The mass spectra of thorium carbonyl ions are shown in Figure 1. The cation spectrum is relatively simple. The most abundant species, as indicated in Figure 1a are Th^+^ and ThO^+^. The peak at *m*/*z=*456 shifts to *m*/*z=*464 in the spectrum from the experiment that uses a ^13^C‐substituted CO sample (see the Supporting Information, Figure S1), which confirms that the observed species corresponds to [Th(CO)_8_]^+^. This is the only homoleptic thorium carbonyl complex observed in the cation mass spectrum. In addition, the mass spectrum also contains ions of the form [OTh(CO)_*n*_]^+^ with *n*=4–6. More peaks are observed in the anion spectrum, as shown in Figure 1b. The spectrum shows a peak at *m*/*z=*456, which corresponds to the mass of [Th(CO)_8_]^−^, and the isotopic shift agrees with the thorium octacarbonyl assignment. Besides [Th(CO)_8_]^−^, the mass spectrum also shows a series of peaks that can be assigned to [ThC_*x*_O_*y*_]^−^ with *x*>*y*. The observation of these anions suggests that some carbon monoxide molecules are dissociated during the pulsed laser ablation‐supersonic expansion process in the ion source.

**Figure 2:**
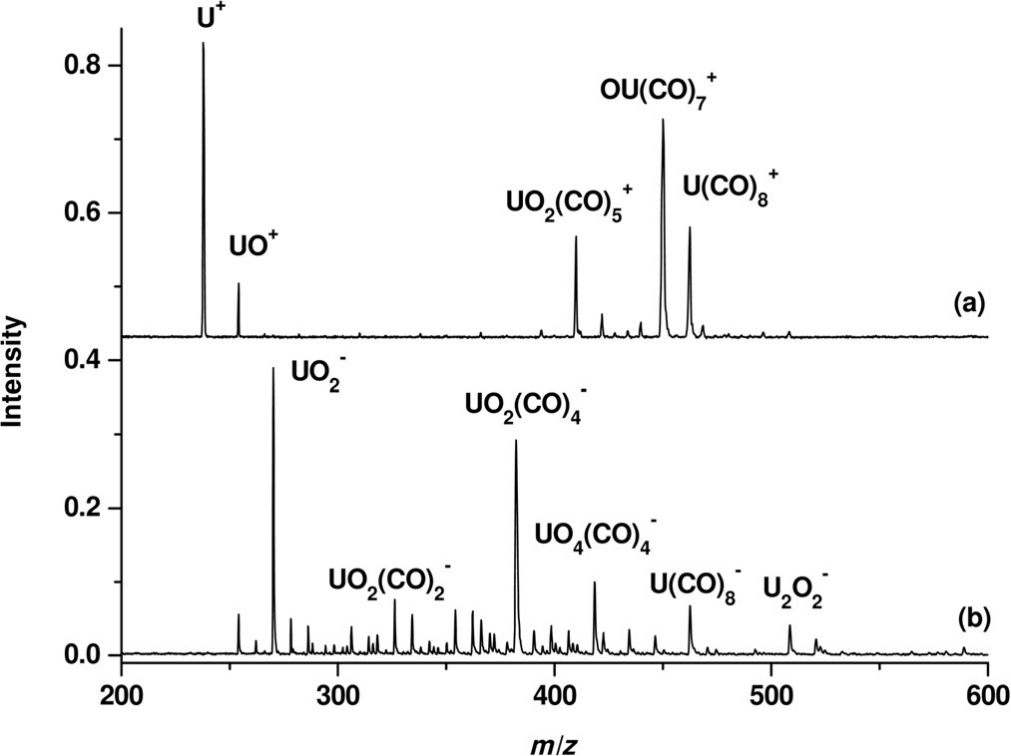
Mass spectra of uranium a) carbonyl cations and b) carbonyl anion complexes.

The mass spectra from the uranium experiments are shown in Figure 2. Besides the strong U^+^ peak, the most abundant species is [OU(CO)_7_]^+^ followed by [U(CO)_8_]^+^ and [UO_2_(CO)_5_]^+^ (2a). Notably, these uranium carbonyl complexes were also observed to be the most abundant species in a previous report.^13^ The anion spectrum is dominated by the peaks that are due to UO_2_^−^ and [UO_2_(CO)_4_]^−^. The [U(CO)_8_]^−^ is also observed with appreciable intensity (Figure 2b).

**Figure 3:**
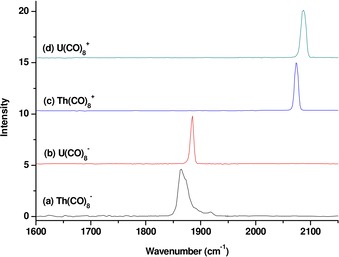
Infrared photodissociation spectra of a) [Th(CO)_8_]^−^, b) [U(CO)_8_]^−^, c) [Th(CO)_8_]^+^, and d) [U(CO)_8_]^+^.

The octacarbonyl anions and cations are mass‐selected for infrared photodissociation. The loss of one CO ligand takes place when the species are excited with unfocused infrared light in the carbonyl stretching frequency region. The infrared spectra are shown in Figure 3. The [Th(CO)_8_]^−^ anion shows two bands at 1865 and 1919 cm^−1^. The [U(CO)_8_]^−^ anion gives only one band at 1885 cm^−1^. These band positions are strongly redshifted with respect to free CO (2143 cm^−1^), which indicates significant metal→CO π backdonation. The [Th(CO)_8_]^+^ and [U(CO)_8_]^+^ cations each display a single band at 2074 and 2087 cm^−1^, respectively. The band positions of the cations are about 200 cm^−1^ blueshifted from those of the anions, but they are still redshifted with respect to free CO. The band position of [U(CO)_8_]^+^ cation is in agreement with the previously reported value of 2080 cm^−1^.^13^ The infrared photodissociation spectra of [Th(^13^CO)_8_]^−^ and [Th(^13^CO)_8_]^+^ are also measured (see the Supporting Information, Figure S2). The band positions are observed at 1827 and 1879 cm^−1^ for the anion and at 2029 cm^−1^ for the cation. The isotopic frequency shifts confirm that these bands are due to carbonyl stretching vibrations.

**Figure 4:**
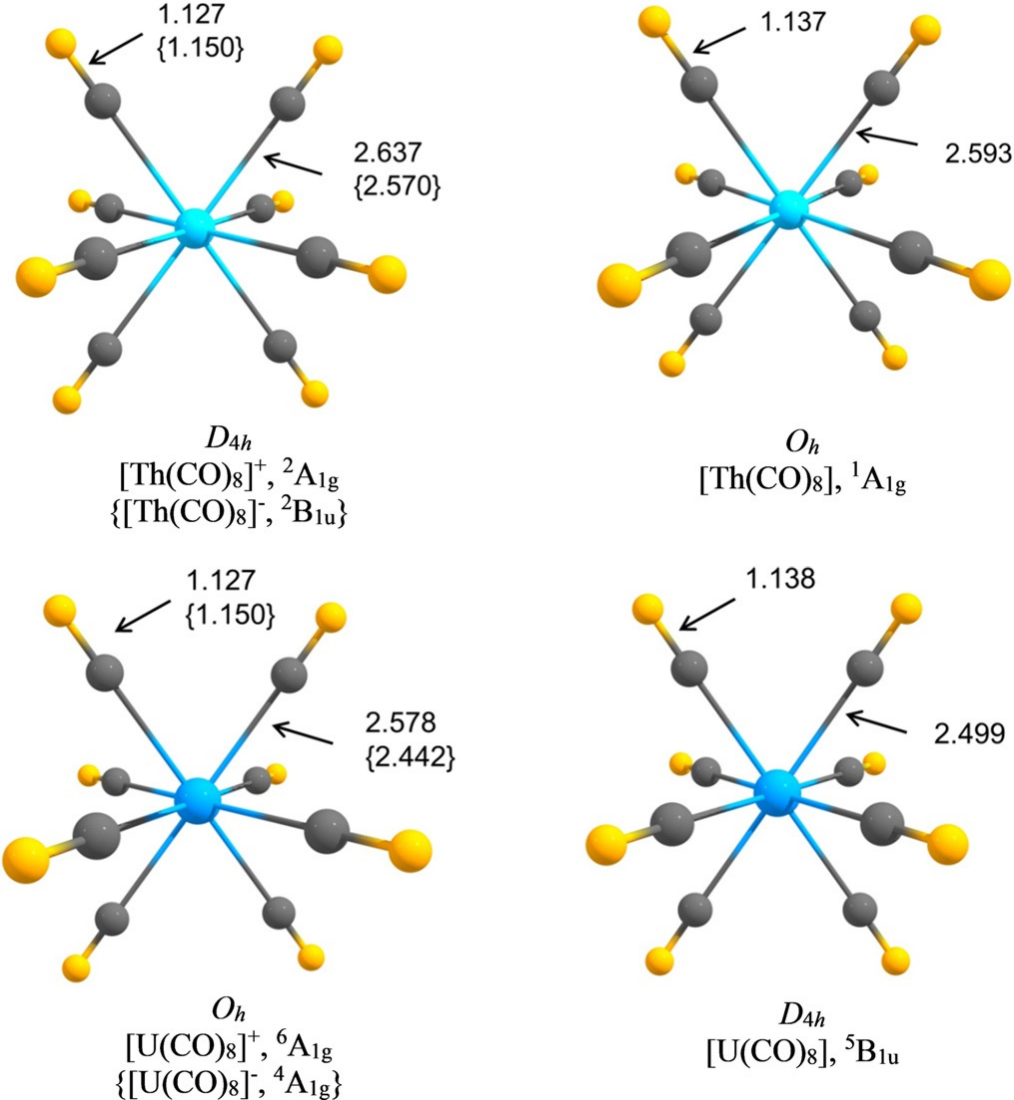
Calculated equilibrium geometries of the octacarbonyl complexes [An(CO)_8_]^*q*^ (*q*=+1, 0, −1; An=Th, U) at the B3LYP‐D3(BJ)/def2‐TZVPPD/ECP level. The bond lengths are given in Å.

Figure 4 shows the calculated equilibrium geometries of the neutral and charged metal complexes [An(CO)_8_]^*q*^ (An=Th, U; *q*=−1, 0, +1) at the B3LYP‐D3(BJ)/def2‐TZVPPD/ECP level (see the Supporting Information, Figures S3 and S4 for the geometries with different spin states and the corresponding relative energies). The thorium octacarbonyl ions possess *D*_4*h*_ symmetry, with the [Th(CO)_8_]^+^ cation having a ^2^A_1g_ electronic ground state and the [Th(CO)_8_]^−^ anion possessing a ^2^B_1u_ ground state. Neutral complex [Th(CO)_8_] has cubic (*O_h_*) symmetry and a singlet (^1^A_1g_) electronic ground state. The uranium octacarbonyl ions also exhibit cubic (*O_h_*) symmetry, with the [U(CO)_8_]^+^ cation having a sextet (^6^A_1g_) electronic ground state and the [U(CO)_8_]^−^ anion possessing a quartet (^4^A_1g_) ground state. Neutral [U(CO)_8_] has *D*_4*h*_ symmetry and a quintet ( ^5^B_1u_) electronic ground state. The calculated C−O distances of all molecules are slightly longer than in free CO (1.125 Å). The different equilibrium geometries of the molecules may be caused by Jahn–Teller distortion. Calculations that used different starting geometries led to the same equilibrium structures.

Table 1 shows the calculated bond dissociation energies (BDEs) for loss of one or all eight CO ligands from the metal octacarbonyl complexes (see the Supporting Information, Figures S5 and S6 for the equilibrium geometries of heptacarbonyl [An(CO)_7_]^*q*^ complexes). The dissociation of one CO ligand from the thorium species [Th(CO)_8_]^*q*^ has very similar values between *D*_e_=20–22 kcal mol^−1^ for the charged and neutral complexes. Nearly the same values are calculated for CO ligand dissociation from cationic [U(CO)_8_]^+^ and neutral [U(CO)_8_] complexes, whereas the anion [U(CO)_8_]^−^ has a slightly larger BDE. The BDEs for loss of one CO from the octacarbonyls are much greater than the energy of the infrared photons in the CO stretching region. Therefore, the experimentally observed dissociation is due to a multiphoton absorption process. The total BDEs of all eight CO ligands are rather high: the values are between 203–245 kcal mol^−1^ for the thorium adducts and 170–249 kcal mol^−1^ for the uranium complexes.

**Table 1:** Calculated bond dissociation energies for the loss of one CO and eight CO ligands from [An(CO)_8_]^*q*^ (*q*=+1, 0, −1) at the B3LYP‐D3(BJ)/def2‐TZVPPD/ECP level. All values are in kcal mol^−1^.^[a]^

Reaction^[a]^	*D*_e_	*D*_0_	Δ*G* ^298 K^
Th(CO)_8_^+^ (*D*_4*h*_, ^2^A_1g_)→Th(CO)_7_^+^ (*C_s_*, ^2^A′)+CO	19.9	18.8	8.5
Th(CO)_8_ (*O_h_*, ^1^A_1g_)→Th(CO)_7_ (*C*_3*v*_, ^1^A_1_)+CO	20.6	19.5	9.1
Th(CO)_8_^−^ (*D*_4*h*_, ^2^B_1u_)→Th(CO)_7_^−^ (*C*_3*v*_, ^2^A_2_)+CO	21.6	20.7	11.6
			
U(CO)_8_^+^ (*O_h_*, ^6^A_1g_)→U(CO)_7_^+^ (*C*_3*v*_, ^6^A_2_)+CO	19.1	17.8	6.9
U(CO)_8_ (*D*_4*h*_, ^5^B_1u_)→U(CO)_7_ (*C_s_*, ^5^A′)+CO	21.2	19.9	9.4
U(CO)_8_^−^ (*O_h_*, ^4^A_1g_)→U(CO)_7_^−^ (*C*_3*v*_, ^4^A_2_)+CO	25.5	24.4	14.0
			
Th(CO)_8_^+^ (*D*_4*h*_, ^2^A_1g_)→′Th^+^ (D)+8 CO	203.2	193.6	123.2
Th(CO)_8_ (*O_h_*, ^1^A_1g_)→Th (T)+8 CO	215.8	205.9	144.3
Th(CO)_8_^−^ (*D*_4*h*_, ^2^B_1u_)→Th^−^ (Q)+8 CO	245.3	236.2	163.9
			
U(CO)_8_^+^ (*O_h_*, ^6^A_1g_)→U^+^ (Q)+8 CO	169.7	160.1	88.5
U(CO)_8_ (*D*_4*h*_, ^5^B_1u_)→U (Q′)+8 CO	192.7	182.8	111.0
U(CO)_8_^−^ (*D*_4*h*_, ^2^B_1u_)→U^−^ (S′)+8 CO	249.3	239.0	164.5

[a] D=doublet, Q=quartet, Q′=quintet; S′=sextet, T=triplet.

Table 2 shows the experimental and calculated wavenumbers for the C−O stretching mode of the thorium and uranium octacarbonyl complexes [An(CO)_8_]^*q*^. There is only one experimentally observed IR active mode of the uranium cation and anion [U(CO)_8_]^*q*^ (*q*=+1, −1) that possesses *O_h_* symmetry, which are redshifted by −56 cm^−1^ for the cation and −258 cm^−1^ for the anion with respect to free CO. This is in excellent agreement with the theoretical values, which suggest a redshift of −63 and −243 cm^−1^, respectively. The agreement between the theoretical and experimental wavenumbers for the C−O stretching mode strongly suggests that the observed species are the uranium complexes [U(CO)_8_]^+^ in the sextet (^6^A_1g_) ground state and the [U(CO)_8_]^−^ anion in the quartet (^4^A_1g_) state. The calculations predict that the uranium octacarbonyl complex [U(CO)_8_], exhibiting *D*_4*h*_ symmetry, has two IR‐active CO stretching modes, which are redshifted by −146 and −153 cm^−1^.

**Table 2:** Calculated^[a]^ (B3LYP‐D3(BJ)/def2‐TZVPPD/ECP) and experimental C−O stretching wavenumbers (cm^−1^) for the [An(CO)_8_]^+/−^ (An=Th, U) ion complexes. Calculated IR intensities (km mol^−1^) are given in parentheses.

	Calcd				Exptl			
	^12^CO	Δ(CO)^[b]^	^13^CO	Δ(CO)^[c]^	^12^CO	Δ(CO)^[b]^	^13^CO	Δ(CO)^[c]^
U(CO)_8_^−^ (*O_h_*, ^4^A_1g_)	2004 (0, a_1g_)	–	–	–	–	–	–	–
	1900 (3873, t_1u_)	−243	–	–	1885	−258	–	–
	1886 (0, a_2u_)	–	–	–	–	–	–	–
	1881 (0, t_2g_)	–	–	–	–	–	–	–
								
U(CO)_8_^+^ (*O_h_*, ^6^A_1g_)	2140 (0, a_1g_)	–	–	–	–	–	–	–
	2080 (1949, t_1u_)	−63	–	–	2087	−56	–	–
	2077 (0, a_2u_)	–	–	–	–	–	–	–
	2067 (0, t_2g_)	–	–	–	–	–	–	–
								
Th(CO)_8_^−^ (*D*_4*h*_, ^2^B_1u_)	2010 (0, a_1g_)	–	1964 (0, a_1g_)	–	–	–	–	–
	1912 (4685, a_2u_)	−231	1870 (4455, a_2u_)	−42	1919	−224	1879	−40
	1894 (0, b_1g_)	–	1851 (0, b_1g_)	–	–	–	–	–
	1886 (3986, e_u_)	−257	1844 (3793, e_u_)	−42	1865	−278	1827	−38
	1884 (0, b_2u_)	–	1841 (0, b_2u_)	–	–	–	–	–
	1851 (0, e_g_)	–	1810 (0, e_g_)	–	–	–	–	–
								
Th(CO)_8_^+^ (*D*_4*h*_, ^2^A_1g_)	2148 (0, a_1g_)	–	2100 (0, a_1g_)	–	–	–	–	–
	2082 (0, b_2u_)	–	2035 (0, b_2u_)	–	–	–	–	–
	2080 (0, b_1g_)	–	2034 (0, b_1g_)	–	–	–	–	–
	2080 (0, e_g_)	–	2034 (0, e_g_)	–	–	–	–	–
	2072 (2528, e_u_)	−71	2026 (2390, e_u_)	−46	2074	−69	2029	−45
	2070 (2355, a_2u_)	−73	2024 (2224, a_2u_)	−46	2074	−69	2029	−45

[a] Scaled by 0.968 by using the ratio of calculated (2213 cm^−1^) and experimental (2143 cm^−1^) wavenumbers of free CO. [b] Frequency shift with respect to free CO. [c] Frequency shift with respect to the ^12^CO isotopomer.

The calculations of the thorium ions [Th(CO)_8_]^*q*^ (*q*=+1, −1) that possess *D*_4*h*_ symmetry predict that there are two IR‐active C−O stretching modes with high intensities. There are two experimental values in the C−O stretching region of the IR spectrum for the anion, but only one signal is observed for the cation (Table 2). However, the calculated IR‐active C−O stretching modes of [Th(CO)_8_]^+^ are only 2 cm^−1^ apart from each other, and thus, they appear as one broad signal. The calculated and experimental wavenumbers for the IR‐active C−O stretching modes of the thorium ions indicate, like the uranium complexes, a redshift with respect to free CO. The very close agreement between the theoretical and the experimental frequency shifts for the ^13^CO isotopes, which were recorded for the thorium species, is strong evidence that the observed species are the [Th(CO)_8_]^+^ cation in the ^2^A_1g_ electronic ground state and the [Th(CO)_8_]^−^ anion in the ^2^B_1u_ state. The calculations predict that the neutral thorium octacarbonyl complex [Th(CO)_8_], having *O_h_* symmetry, exhibits a single IR‐active stretching mode, which is redshifted by −149 cm^−1^.

The DFT results come from single‐reference calculations and the accuracy of the computed data may be questioned. CASSCF calculations that use the full valence space are not possible owing to the size of the active space of the metal octacarbonyl complexes. There are five d and seven f AOs of the metal and one σ and two π MOs of the carbonyl ligands, which give an active space of 36 orbitals that are occupied by 19 (Th(CO)_8_^+^) to 23 electrons (U(CO)_8_^−^). We carried out CCSD(T)/def2‐SVP/ECP calculations of the uranium and thorium octacarbonyl complexes by using the B3LYP‐D3(BJ)/def2‐TZVPPD/ECP optimized geometries. The *T*_1_ diagnostics^33^, ^34^ indicate that all species can reliably be calculated with single‐reference methods. The calculated *T*_1_ values are given in Table S1 (see the Supporting Information), and the relative energies of the different electronic states at CCSD(T)/def2‐SVP/ECP are shown in Figures S3 and S4 (see the Supporting Information). The ab initio calculations give the same electronic states as the lowest energy form in the DFT calculations, except for cation U(CO)_8_^+^. The uranium octacarbonyl cation is predicted at CCSD(T)/def2‐SVP/ECP to possess a *D*_2*h*_ structure and an electronic quartet state. Calculations with a larger basis set at CCSD(T)/def2‐TZVP/ECP also give a quartet state to be lower in energy than the sextet. It was already reported in an earlier DFT study by Ricks et al. that the electronic sextet and quartet state of U(CO)_8_^+^ cation are close in energy and that an accurate calculation should include spin‐orbit coupling.^13^ Geometry optimization of U(CO)_8_^+^ cation at high‐level ab initio methods with the inclusion of spin‐orbit coupling are not possible by us. The experimentally observed vibrational spectrum is likely to come from the electronic sextet state of the U(CO)_8_^+^ cation with *O_h_* geometry. The calculated IR spectrum of the quartet state shows two strongly IR‐active signals that are redshifted by 63 and 71–73 cm^−1^ and are separated by 10 cm^−1^ (see the Supporting Information, Table S2). The observed spectrum shows only one sharp signal (Figure 3). We, therefore, think that the U(CO)_8_^+^ cation has a sextet ground state (^6^A_1g_).

We theoretically analyzed the metal–CO bonds of the octacarbonyl complexes with the EDA‐NOCV method to gain insights into the nature of the interatomic interactions. A central question of the bonding analysis concerns the contributions of the metal valence orbitals to the bonds. The EDA‐NOCV method is well suited for this, because it uses the electronic structure of the molecules for an unbiased analysis of the pairwise orbital interactions without referring to a reference system. This has been found very useful in our previous studies of metal octacarbonyl complexes of main‐group atoms,^17^ transition metals,^16^ and late‐lanthanide elements.^10^ A focus of the present work lies on the contribution of the 5f orbitals of the metals to the metal–CO bonds. Our previous bonding analysis of the lanthanide octacarbonyl anions [Ln(CO)_8_]^−^ (Ln=Tm, Yb, Lu) showed that the 4f orbitals of the Ln atoms provide <4 % of the total orbital interactions of the Ln^−^−CO bonds. The dominant covalent bonding comes from Ln(d)^−^→CO π backdonation and Ln(d)^−^←CO σ donation, which account for >90 % of the orbital interaction.^10^ It is interesting to compare these results with the present study of the octacarbonyl complexes of the 5f elements thorium and uranium.

**Figure 5:**
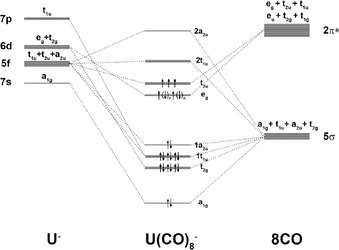
Splitting of the 5f, 6d, 7s, and 7p valence orbitals of uranium in the cubic (*O_h_*) field of eight CO ligands and interactions with the 5σ and 2π* valence MOs of (CO)_8_. The e_g_ orbital is fully occupied (*n*=1) in the quartet (^4^A_1g_) state of the anion [U(CO)_8_]^−^. It is occupied with two unpaired electrons (*n*=0) in the sextet (^6^A_1g_) state of the cation [U(CO)_8_]^+^. The MOs of U(CO)_8_^+/−^ show the ordering of the calculated highest‐lying occupied and lowest‐lying vacant orbitals.

The bonding analysis of the [An(CO)_8_]^*q*^ complexes is simplified by the high symmetry of the structures, because the metal AOs split according to the irreducible representation of the point group. Figure 5 shows the orbital correlation diagram for uranium ion U^*q*^ with 7s, 7p, 6d, and 5f valence orbitals and eight CO in the cubic (*O_h_*) field of [U(CO)_8_]^*q*^. The e_g_ MO is fully occupied in the quartet (^4^A_1g_) state of the anion [U(CO)_8_]^−^, whereas it is occupied with two unpaired electrons in the sextet (^6^A_1g_) state of the cation [U(CO)_8_]^+^. It becomes obvious that the electronic reference configuration of U^+^ in the sextet state is 7s^0^ 7p^0^ 6d^2^ 5f^3^, whereas the quartet state of U^−^ has the electron configuration 7s^0^ 7p^0^ 6d^4^ 5f^3^. The AOs of the uranium atom uniquely correlate with the MOs of the complexes [U(CO)_8_]^*q*^, except for the t_1u_ component of the 5f AOs, which can mix with the t_1u_ set of the 7p AOs. However, inspection of the deformation densities, which are provided by the EDA‐NOCV calculations, makes it possible to estimate the relative contributions of the metal 5f and 7p AOs to the t_1u_ MO. This is discussed below.

**Figure 6:**
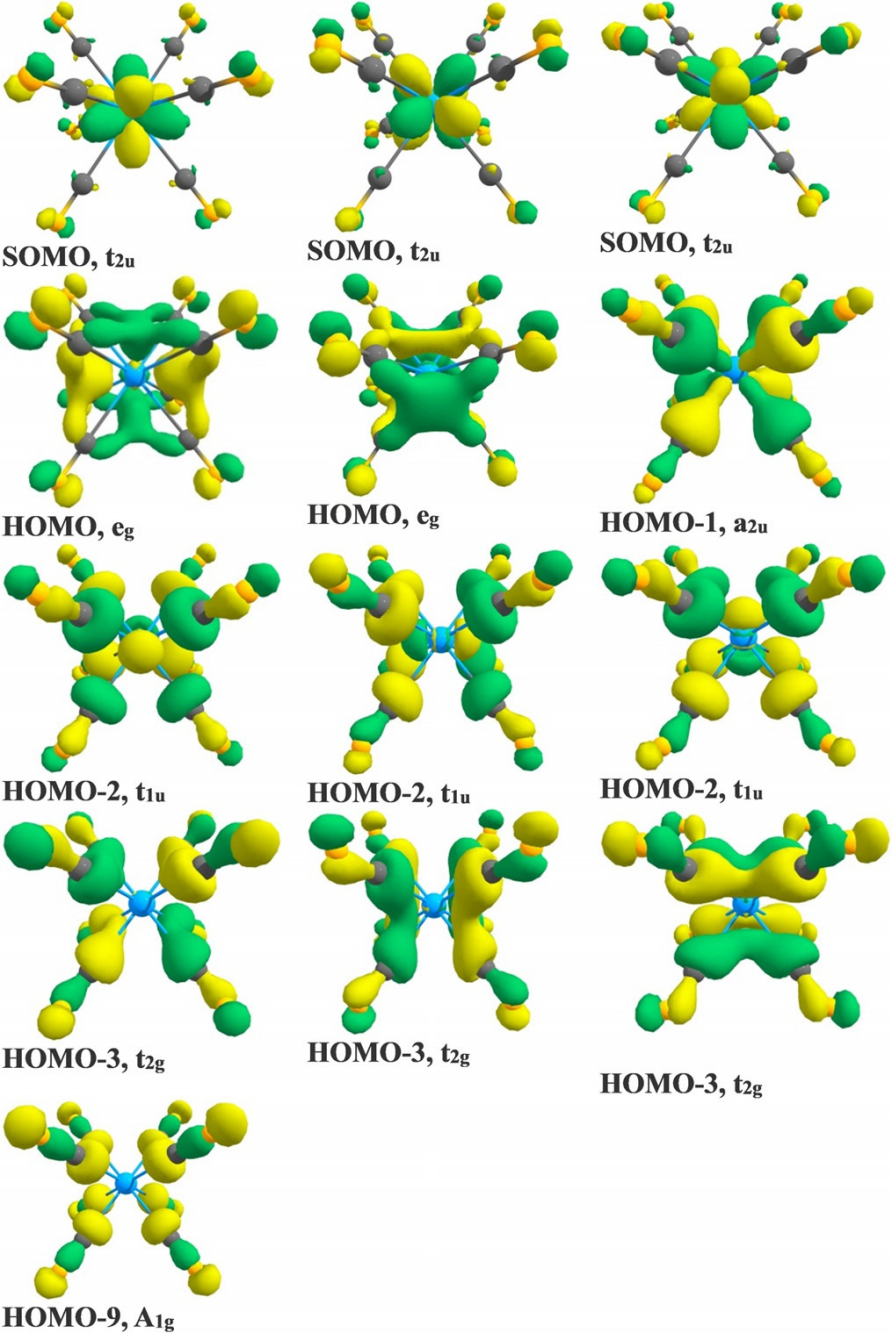
Contour isosurfaces (0.03 a.u.) of the Kohn–Sham molecular orbitals of (^4^A_1g_) [U(CO)_8_]^−^ at the B3LYP‐D3(BJ)/def2‐TZVPPD/ECP level showing U−CO bonding.

Figure 6 shows a plot of the occupied U−CO bonding MOs of [U(CO)_8_]^−^, which correlate with the orbitals in the correlation diagram of Figure 5. It becomes obvious that the degenerate set of singly occupied t_2u_ orbitals and the a_2u_ MO have some contributions from the 5f AOs of uranium, but the relevance of the 5f‐orbital bonding to the metal–CO bonds is unclear. A quantitative estimate of the contribution of the 5f AOs to the bonding interactions is provided by the EDA‐NOCV method. Table 3 shows the numerical results of the EDA‐NOCV calculations of the uranium octacarbonyls [U(CO)_8_]^*q*^ (*q*=+1, −1) by using the metal ion U^*q*^ with the given electron configurations and (CO)_8_ as interacting fragments, which refer to the MO correlation diagram.

**Table 3:** EDA‐NOCV results for (X^6^A_1g_) [U(CO)_8_]^+^ and (X^4^A_1g_) [U(CO)_8_]^−^ complexes at the B3LYP‐D3(BJ)/TZ2P level by using the B3LYP‐D3(BJ)/def2‐TZVPPD/ECP optimized geometries. All values in kcal mol^−1^.

Energy terms	Orbital interaction	[U(CO)_8_]^+^	[U(CO)_8_]^−^
Fragments		U^+^ Sextet (7s^0^ 7p^0^ 6d^2^ 5f^3^) +(CO)_8_ (Singlet)	U^−^ Quartet (7s^0^ 7p^0^ 6d^4^ 5f^3^)+(CO)_8_ (Singlet)
Δ*E*_int_	–	−202.9	−366.8
Δ*E*_Pauli_	–	247.4	415.8
Δ*E*_disp_^[a]^	–	−10.3 (2.3 %)	−10.2 (1.3 %)
Δ*E*_elstat_^[a]^	–	−189.6 (42.1 %)	−307.8 (39.3 %)
Δ*E*_orb_^[a]^	–	−250.3 (55.6 %)	−464.7 (59.4 %)
Δ*E*_orb(1)_^[b,c]^ (e_g_)	[U(d)]^*q*^→(CO)_8_ π backdonation	−80.1 (32.0 %)	−286.1 (61.6 %)
Δ*E*_orb(2)_^[b,c]^ (t_2g_)	[U(d)]^*q*^←(CO)_8_ σ donation	−80.1 (32.0 %)	−81.0 (17.4 %)
*E*_orb(3)_^[b,c]^ (t_2u_)	[U(f)]^*q*^→(CO)_8_ π backdonation	−22.0 (8.8 %)	−52.0 (11.2 %)
Δ*E*_orb(4)_^[b]^ (a_2u_)	[U(f)]^*q*^←(CO)_8_ σ donation	−19.1 (7.6 %)	−21.3 (4.6 %)
Δ*E*_orb(5)_^[b]^ (a_1g_)	[U(s)]^*q*^←(CO)_8_ σ donation	−9.0 (3.6 %)	−4.4 (0.9 %)
Δ*E*_orb(6)_^[b,c]^ (t_1u_)	[U(p,f)]^*q*^←(CO)_8_ σ donation	−12.3 (4.9 %)	−5.7 (1.2 %)
Δ*E*_orb(rest)_^[b]^	–	−27.7 (11.1 %)	−14.2 (3.1 %)

[a] The values within the parentheses show the contribution to the total attractive interactions Δ*E*_elstat_+Δ*E*_orb_+Δ*E*_disp_. [b] The values within the parentheses show the contribution to the total orbital interaction, Δ*E*_orb_. [c] The sum of the two (e_g_) or three (t_2g_, t_1u_, t_2u_) components is given.

The data in Table 3 suggest that the intrinsic interactions between the uranium ion and the octacarbonyl ligands have a slightly higher covalent than electrostatic character. The covalent orbital term Δ*E*_orb_ contributes 56 % to the total attraction in [U(CO)_8_]^+^ and 59 % in [U(CO)_8_]^−^. The most important information of the EDA‐NOCV calculations comes from the breakdown of Δ*E*_orb_ into the pairwise orbital interactions of Δ*E*_orb(1)_−Δ*E*_orb(6)_. The contribution of the [U(d)]^+^→(CO)_8_ π backdonation and [U(d)]^+^←(CO)_8_ σ donation in the [U(CO)_8_]^+^ cation have the same strength; they amount to 64 % of Δ*E*_orb_. Not surprisingly, the [U(d)]^−^→(CO)_8_ π backdonation in the [U(CO)_8_]^−^ anion is much stronger than the [U(d)]^−^←(CO)_8_ σ donation; both comprise 79 % of the total orbital interactions. Thus, the valence d AOs are the most important metal orbitals in [U(CO)_8_]^*q*^, but the relative contribution to the metal–ligand bonding is smaller than in the late lanthanide octacarbonyl anions [Ln(CO)_8_]^−^ (Ln=Tm, Yb, Lu), in which the metal d‐orbital interactions account for >90 % of the metal‐AO contribution to the covalent bonding.^10^

Table 3 shows that the orbital interactions that come from the [U(f)]^*q*^→(CO)_8_ π backdonation and the [U(f)]^*q*^←(CO)_8_ σ donation contribute 16 % to the total orbital interactions in the cation and in the anion [U(CO)_8_]^*q*^. The data suggest a significant contribution of the metal 5f orbitals to the chemical bonds. The contribution of the 5f AOs to the t_1u_ orbitals, which have mainly 6p character at the metal side, appears to be small. This becomes obvious from the visual inspection of the deformation densities and the associated fragment orbital, which are shown below. The contribution of the uranium 5f AOs to the U−CO bonds in [U(CO)_8_]^*q*^ is much higher than that of the 4f AOs of the lanthanide atoms in [Ln(CO)_8_]^−^ (Ln=Tm, Yb, Lu), which amount to <4 % of the Ln−CO bonding orbitals.^10^ The EDA‐NOCV results of Table 3 suggest that the contribution of the uranium valence orbitals possess the trend 6d≫5f>7s≈7p. We want to point out that the Δ*E*_orb_ values do not only come from genuine orbital interactions between the fragments, they also include intrafragment polarization. A previous study of transition metal hexacarbonyls [TM(CO)_6_]^*q*^ (TM^*q*^=Hf^2−^, Ta^−^, W, Re^+^, Os^2+^, Ir^3+^) showed that the contribution by polarization is rather small.7c It can be assumed that it does not change the trend of the actinide valence orbitals in the chemical bonds of [An(CO)_8_]^*q*^.

**Figure 7:**
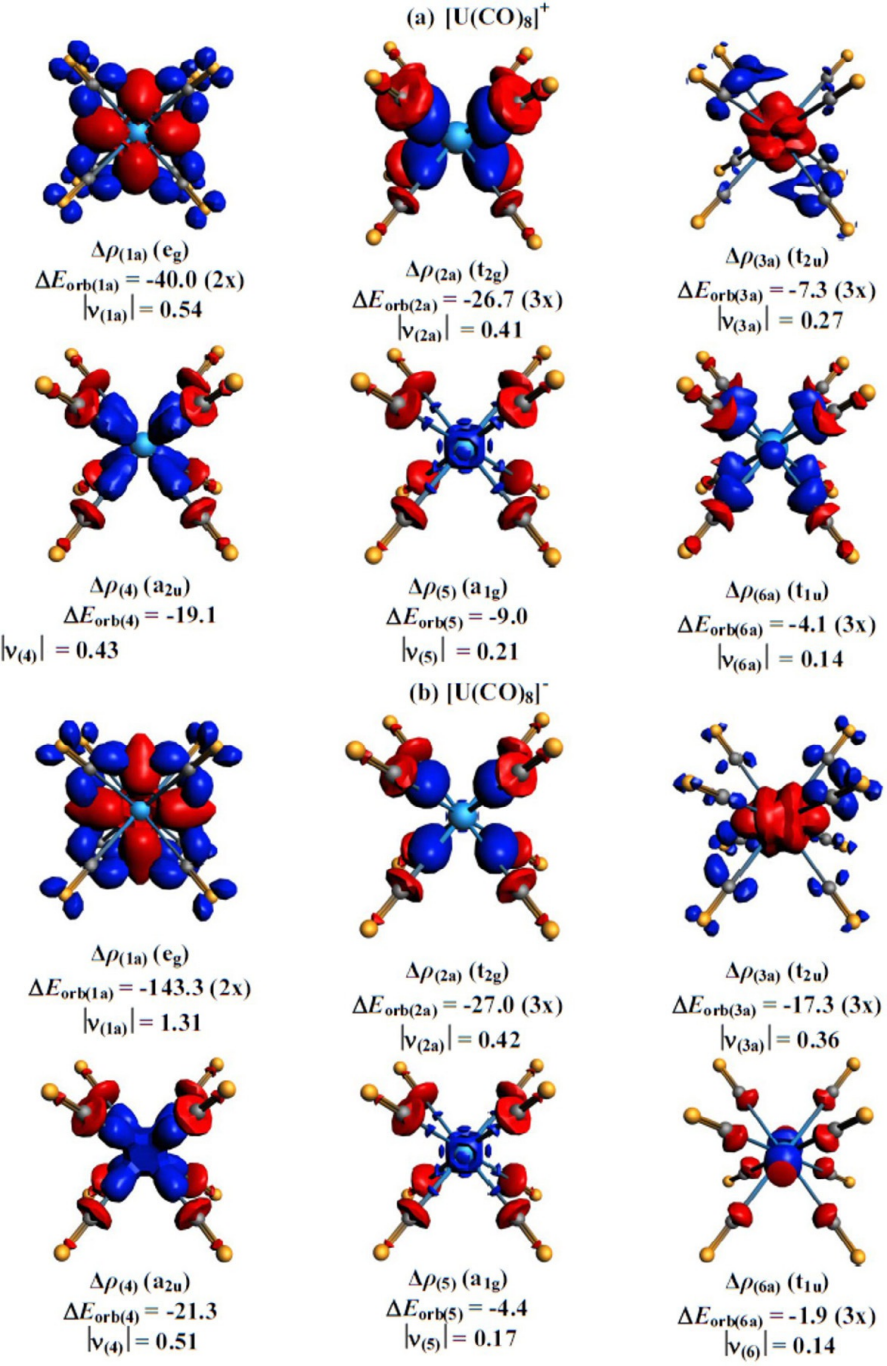
Plot of the deformation densities Δ*ρ*_(1)_−Δ*ρ*_(6)_, which are associated with the individual components of the orbital interactions Δ*E*_orb(1)_−Δ*E*_orb(6)_ in a) [U(CO)_8_]^+^ and b) [U(CO)_8_]^−^ by using U^*q*^ and (CO)_8_ as the interacting fragments (see Table 3). Only one component of the degenerate orbitals is shown. The color code of the charge flow is red→blue. Energy values are given in kcal mol^−1^. The eigenvalues |*ν*| indicate the size of the charge migration. For [U(CO)_8_]^+^, the isosurface values are 0.001 for Δ*ρ*_(1)_−Δ*ρ*_(4)_ and 0.0008 for Δ*ρ*_(5)_ and Δ*ρ*_(6)_. For [U(CO)_8_]^−^, the isosurface values are 0.002 for Δ*ρ*_(1)_, 0.001 for Δ*ρ*_(2)_−Δ*ρ*_(4)_, and 0.0008 for Δ*ρ*_(5)_ and Δ*ρ*_(6)_.

Figure 7 shows the plots of the deformation densities Δ*ρ*_(1)_−Δ*ρ*_(6)_, which are associated with the orbital interactions Δ*E*_orb(1)_−Δ*E*_orb(6)_ in [U(CO)_8_]^+^ and [U(CO)_8_]^−^. Only one component of the degenerate interactions is shown. The color code of the charge flow is red→blue. Note that the plots of the deformation densities of the weaker interactions have smaller isovalues than those of the stronger interactions. The deformation densities nicely show the charge flow that is due to U^*q*^←(CO)_8_ σ donation into the vacant 6d (t_2g_) and 5f AOs (a_2u_) and the reverse U^*q*^→(CO)_8_ π backdonation from the occupied 6d (e_g_) and 5f AOs (t_2u_). Inspection of the deformation density Δ*ρ*_(6a)_ (t_1u_) reveals that the uranium orbital is mainly a 7p AO. The occurrence of a red and a blue area in the deformation density Δ*ρ*_(6a)_ of the anion [U(CO)_8_]^−^ reveals a hybridization 5f/7p at the uranium center during bond formation. Figure 7 gives also the eigenvalues |*ν*|, which indicate the size of the charge transfer associated with the respective orbital interactions. Note that the amount of relocated charge does not directly correspond to the energy, because it is also determined by the energy levels of the interaction orbitals.

**Figure 8:**
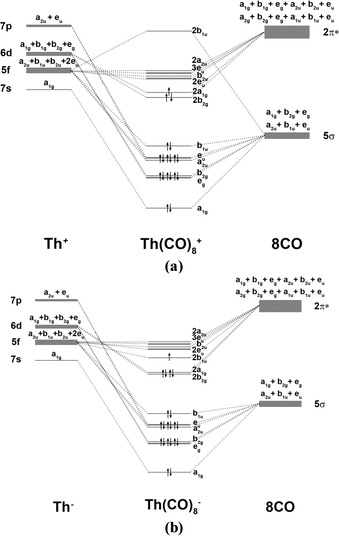
Splitting of the 5f, 6d, 7s, and 7p valence orbitals of thorium in the *D*_4*h*_ field of eight CO ligands and interactions with the 5σ and 2π* valence MOs of (CO)_8_ in a) octacarbonyl cation [Th(CO)_8_]^+^ and b) octacarbonyl anion [Th(CO)_8_]^−^. The MOs of Th(CO)_8_^+/−^ show the ordering of the calculated highest‐lying occupied and lowest‐lying vacant orbitals.

Figure 8 shows the orbital correlation diagrams between the metal valence AOs and eight CO ligands for the thorium cation and anion complexes [Th(CO)_8_]^+^ and [Th(CO)_8_]^−^ by using the *D*_4*h*_ symmetry of the equilibrium structures. They look more complicated than the MO diagram for the *O_h_* field of the uranium adducts (Figure 5), but the splittings are related to each other. The triply degenerate orbitals of the *O_h_* field split into doubly degenerated orbitals and one non‐degenerate orbital as follows: t_1u_(*O_h_*)→e_u_+a_2u_(*D*_4*h*_); t_2u_(*O_h_*)→e_u_+b_2u_(*D*_4*h*_); t_2g_(*O_h_*)→e_g_+b_2g_(*D*_4*h*_). The doubly degenerate orbitals are split as e_g_(*O_h_*)→a_1g_+b_1g_(*D*_4*h*_). Furthermore, a_2u_(*O_h_*)→b_1u_(*D*_4*h*_).^35^ The orbital correlation diagram suggests that the atomic reference configurations of thorium are 7s^0^ 7p^0^ 6d^3^ 5f^0^ for the doublet state of Th^+^ in (^2^A_1g_) [Th(CO)_8_]^+^ and 7s^0^ 7p^0^ 6d^4^ 5f^1^ for the doublet state of Th^−^ in (^2^B_1u_) [Th(CO)_8_]^−^. Thus, the irreducible representations of the *D*_4*h*_ point group make it possible to distinguish between the contributions of the metal valence AOs, except for the 5f and 7p participation in the e_u_ and a_2u_ MOs, which require inspection of the associated deformation densities.

**Figure 9:**
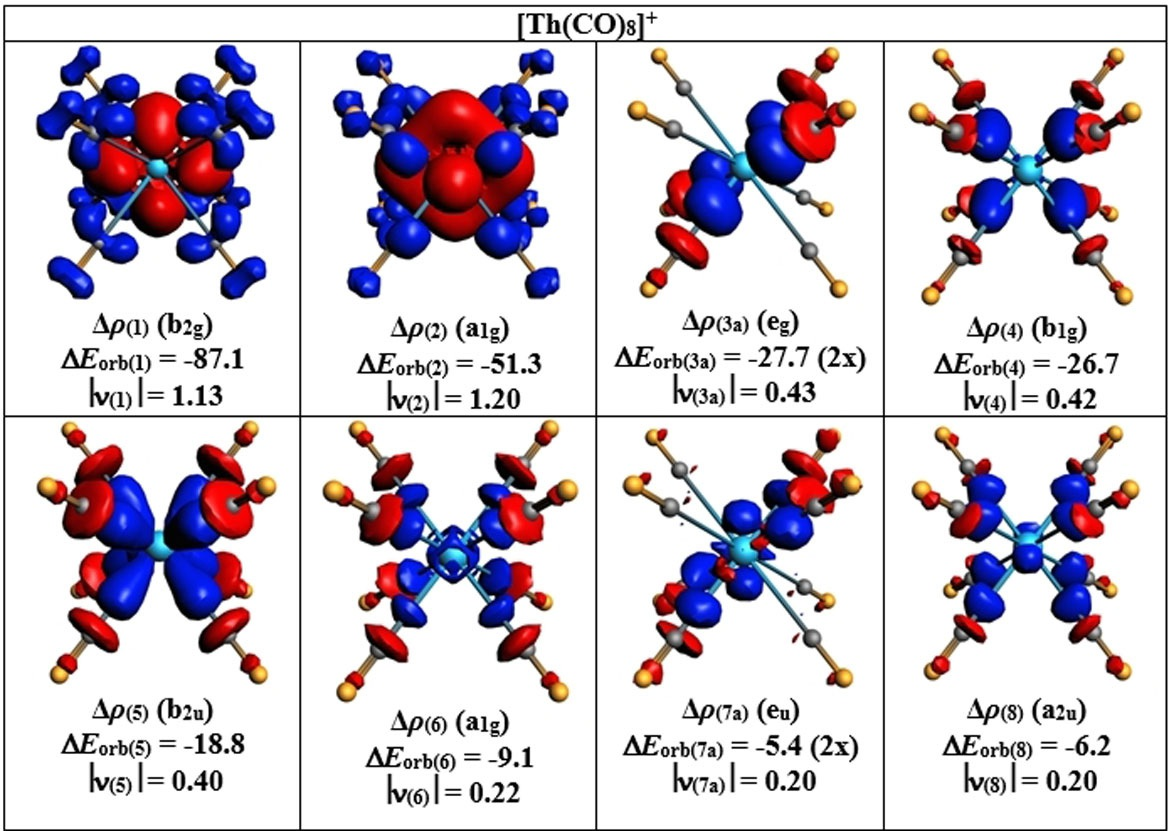
Plot of the deformation densities Δ*ρ*_(1)_−Δ*ρ*_(8)_, which are associated with the individual components of the orbital interactions Δ*E*_orb(1)_−Δ*E*_orb(8)_ in [Th(CO)_8_]^+^ by using Th^+^ and (CO)_8_ as the interacting fragments (Table 4). Only one component of the degenerate orbitals is shown. The color code of the charge flow is red→blue. Energy values are given in kcal mol^−1^. The eigenvalues |*ν*| indicate the size of the charge migration. The isosurface values are 0.002 for Δ*ρ*_(1)_, 0.001 for Δ*ρ*_(2)_−Δ*ρ*_(5)_, and 0.0008 for Δ*ρ*_(6)_−Δ*ρ*_(8)_.

Table 4 shows the numerical results of the EDA‐NOCV calculations of [Th(CO)_8_]^+^ by using the metal ion Th^+^ and the (CO)_8_ cage with the symmetry‐adapted electron configurations (Figure 8) as interacting fragments. The Th^*q*^‐(CO)_8_ interactions possess, like the uranium complexes, a slightly more covalent than electrostatic character. The most important metal AOs in the thorium complex are the 6d orbitals, which have an even higher contribution to the metal–CO bonds than in the uranium complexes. The dominant orbital interactions in the cation [Th(CO)_8_]^+^ come from the [Th(d)]^+^→(CO)_8_ π backdonation (Δ*E*_orb(1)_+Δ*E*_orb(2)_) followed by the [Th(d)]^+^←(CO)_8_ σ donation (Δ*E*_orb(3)_+Δ*E*_orb(4)_). The contribution of the metal(6d) AOs to the bonding in [Th(CO)_8_]^+^ (75 %) is even higher than in [U(CO)_8_]^+^. Inspection of the deformation densities of the e_u_ orbital (Δ*E*_orb(7)_) at the metal end shows that it comes mainly from the 5f AOs, whereas the a_2u_ orbital (Δ*E*_orb(8)_) comes mainly from the 7p AOs. The total contribution of the metal(5f) AOs in [Th(CO)_8_]^+^, which arises from Δ*E*_orb(5)_ and Δ*E*_orb(7)_, amounts to ≈10 % and is less than in [U(CO)_8_]^+^. The EDA‐NOCV results suggest that the contribution of the dominant pairwise orbital interaction in [Th(CO)_8_]^+^ has the same trend as the uranium complexes 6d≫5f>7s≈7p. Figure 9 shows the deformation densities Δ*ρ*_(1)–(8)_ that are associated with the interaction energies Δ*E*_orb(1)–(8)_ in [Th(CO)_8_]^+^. They nicely illustrate the charge flow between the metal atom and the ligand orbitals. Only one component of the degenerate orbital is shown.

**Table 4:** EDA‐NOCV results for (^2^A_1g_) [Th(CO)_8_]^+^ and (X^2^B_1u_) [Th(CO)_8_]^−^ complexes at the B3LYP‐D3(BJ)/TZ2P level by using the B3LYP‐D3(BJ)/def2‐TZVPPD/ECP optimized geometries. All values in kcal mol^−1^.

Energy terms	Orbital interaction	[Th(CO)_8_]^+^	[Th(CO)_8_]^−^	
Fragments		Th^+^ Doublet (7s^0^ 6d^3^ 5f^0^)+(CO)_8_	Th^−^ Doublet (7s^0^ 6d^4^ 5f^1^)+(CO)_8_	Th Singlet (7s^0^ 6d^4^ 5f^0^)+(CO)_8_^−^
Δ*E*_int_	–	−255.1	−387.2	−338.7
Δ*E*_Pauli_	–	248.0	329.8	284.3
Δ*E*_disp_^[a]^	–	−13.7 (2.7 %)	−13.6 (1.9 %)	−13.6 (2.2 %)
Δ*E*_elstat_^[a]^	–	−195.2 (38.8 %)	−259.8 (36.2 %)	−237.4 (38.1 %)
Δ*E*_orb_^[a]^	–	−294.3 (58.5 %)	−443.6 (61.9 %)	−372.0 (59.7 %)
Δ*E*_orb(1)_^[b]^ (2b_2g_)	[Th(d)]^*q*^→(CO)_8_^*q*^ π backdonation	−87.1 (29.6 %)	−125.7 (28.3 %)	−120.1 (32.3 %)
Δ*E*_orb(2)_^[b]^ (2a_1g_)	[Th(d)]^*q*^→(CO)_8_^*q*^ π backdonation	−51.3 (17.4 %)	−129.0 (29.1 %)	−121.0 (32.5 %)
Δ*E*_orb(3)_^[b,c]^ (e_g_)	[Th(d)]^*q*^←(CO)_8_^*q*^ σ donation	−55.4 (18.8 %)	−41.6 (9.4 %)	−49.0 (13.2 %)
Δ*E*_orb(4)_^[b]^ (b_2g_)	[Th(d)]^*q*^←(CO)_8_^*q*^ σ donation	−26.7 (9.1 %)	−19.2 (4.3 %)	−22.8 (6.1 %)
Δ*E*_orb(5)_^[b]^ (b_1u_)	[Th(f)]^*q*^←(CO)_8_^*q*^ σ donation	−18.8 (6.4 %)	−13.4 (3.0 %)	−17.0 (4.6 %)
Δ*E*_orb(6)_^[b]^ (a_1g_)	[Th(s)]^*q*^←(CO)_8_^*q*^ σ donation	−9.1 (3.1 %)	−4.3 (1.0 %)	−6.0 (1.6 %)
Δ*E*_orb(7)_^[b,c]^ (e_u_)	[Th(p,f)]^*q*^←(CO)_8_^*q*^ σ donation	−10.8 (3.7 %)	−6.4 (1.4 %)	−9.6 (2.6 %)
Δ*E*_orb(8)_^[b]^ (a_2u_)	[Th(p,f)]^*q*^←(CO)_8_^*q*^ σ donation	−6.2 (2.1 %)	−1.5 (0.3 %)	−2.4 (0.6 %)
Δ*E*_orb(9)_^[b]^ (2b_1u_)	[Th(f)]^*q*^→(CO)_8_^*q*^ π backdonation	–	−92.1 (20.8 %)	–
Δ*E*_orb(9′)_^[b]^ (2b_1u_)	[Th(f)]^*q*^←(CO)_8_^*q*^ π donation	–	–	−9.4 (2.5 %)
Δ*E*_orb(rest)_^[b]^	–	−28.9 (9.8 %)	−10.4 (2.3 %)	−14.7 (4.0 %)

[a] The values in parentheses show the contribution to the total attractive interactions Δ*E*_elstat_+Δ*E*_orb_+Δ*E*_disp_. [b] The values in parentheses show the contribution to the total orbital interaction, Δ*E*_orb_. [c] The sum of the two (e_g_, e_u_) components is given.

**Figure 10:**
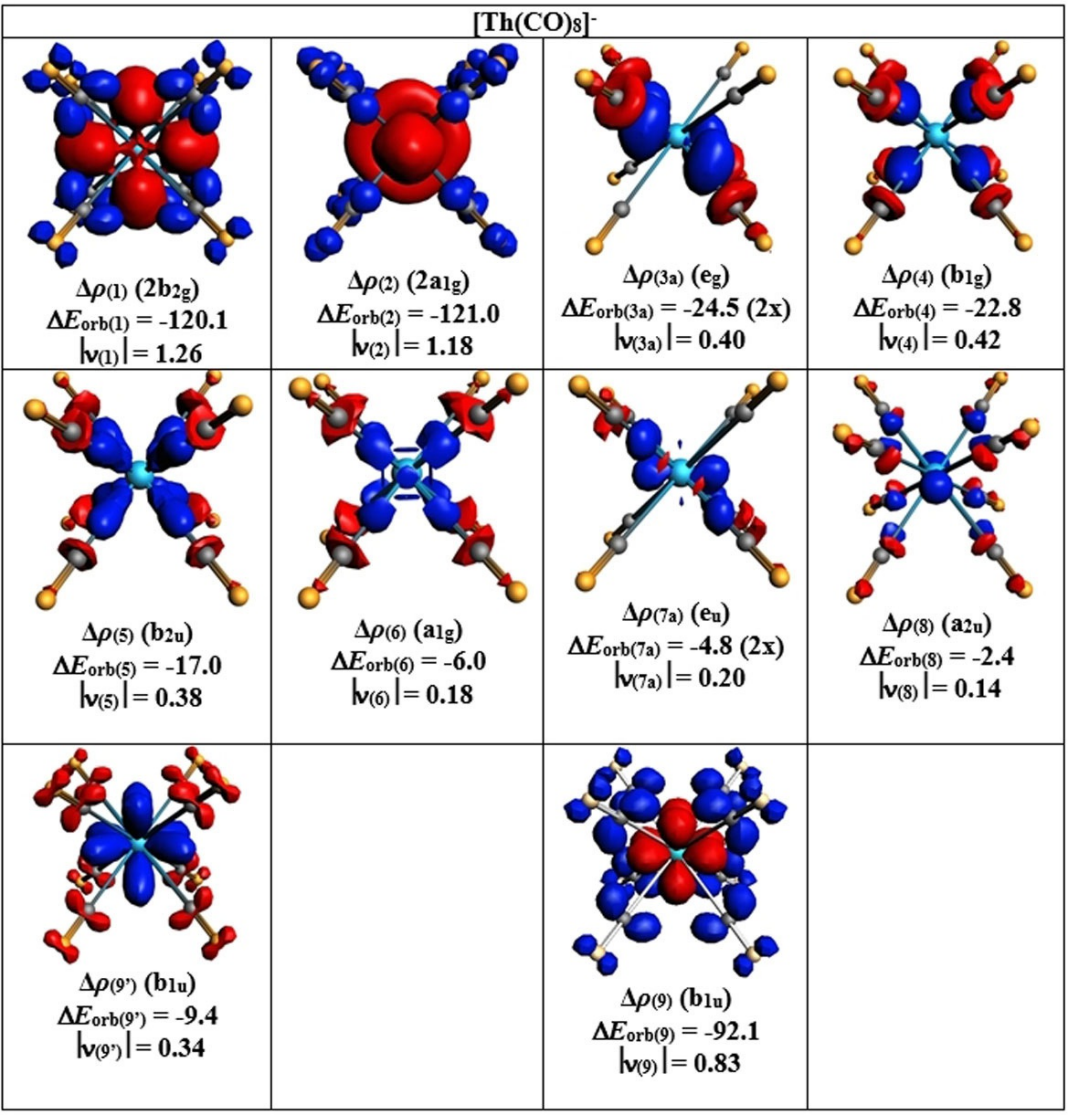
Plot of the deformation densities Δ*ρ*_(1)_−Δ*ρ*_(9**′**)_, which are associated with the individual components of the orbital interactions Δ*E*_orb(1)_−Δ*E*_orb(9**′**)_ in [Th(CO)_8_]^−^ by using Th and (CO)_8_^−^ as the interacting fragments. The deformation density Δ*ρ*_(9)_ comes from the interactions between Th^−^ and (CO)_8_ (Table 4). Only one component of the degenerate orbitals is shown. The color code of the charge flow is red→blue. Energy values are given in kcal mol^−1^. The eigenvalues |*ν*| indicate the size of the charge migration. The isosurface values are 0.003 for Δ*ρ*_(1)_ and Δ*ρ*_(2)_ and 0.001 for others.

The EDA‐NOCV results for the anion [Th(CO)_8_]^−^ in Table 4 are intriguing. The calculation that uses the fragments Th^−^ (D; 7s^0^ 6d^4^ 5f^1^) and (CO)_8_, which correspond to the MO correlation diagram in Figure 8, gives an unusually high contribution from the singly occupied 2b_1u_ MO and suggests strong [Th(f)]^−^→(CO)_8_ π backdonation (Δ*E*_orb(9)_). Inspection of the eigenvalues |*ν*| associated with the deformation densities, which are shown in Figure 10, suggests that the single electron is located more on the ligand cage (CO)_8_ than at the metal atom. This means that the metal–ligand interactions in [Th(CO)_8_]^−^ might be better described in terms of interactions between neutral Th and a negatively charged ligand cage (CO)_8_^−^, although the bond cleavage gives Th^−^ and 8 CO as fragments.^36^

Table 4 shows also the EDA‐NOCV calculations of [Th(CO)_8_]^−^ by using neutral Th, which is in the electronic singlet state and the electron configuration 7s^0^ 6d^4^ 5f^0^, and (CO)_8_^−^ as interacting fragments. Previous studies by us suggest that those fragments that give the smallest orbital value Δ*E*_orb_ are best suited to describe the bond formation, because they undergo the least change by the interatomic interactions.^37^ The results in Table 4 show that the choice of Th+(CO)_8_^−^ as fragments gives a clearly smaller Δ*E*_orb_ value (−237.4 kcal mol^−1^) than Th^−^+(CO)_8_ (−259.8 kcal mol^−1^). Thus, the former EDA‐NOCV calculations provide a more faithful indication of the orbital interactions than the latter. Table 4 shows that the contribution of the singly occupied 2b_1u_ MO, which now comes from [Th(f)]←(CO)_8_^−^ π donation (Δ*E*_orb(9′)_), is much weaker than the [Th(f)]^−^→(CO)_8_ π backdonation (Δ*E*_orb(9)_). The remaining contributions all become a bit larger when Th+(CO)_8_^−^ are employed as the fragments. The latter EDA‐NOCV calculations of the anion suggest a similar result as for the thorium cation, in other words, there is a dominant contribution of the 6d metal orbitals (84 %), which is larger than in the uranium complex. The contribution of the 5f AOs from the b_1u_ orbitals Δ*E*_orb(5)_+Δ*E*_orb(9′)_ and the e_u_ MO Δ*E*_orb(7)_, which has mainly 5f character at the metal end, amounts to ≈9 %. The overall relevance of the thorium valence AOs in [Th(CO)_8_]^−^ has the same trend as the other systems 6d≫5f>7s≈7p.

Figure 10 shows the deformation densities Δ*ρ*_(1)–(9′)_ that are associated with the interaction energies Δ*E*_orb(1)–(9′)_ in [Th(CO)_8_]^−^ by using neutral Th (S, 7s^0^ 6d^4^ 5f^0^) and (CO)_8_^−^ as interacting fragments. We also show the deformation density Δ*ρ*_(9)_ that comes from the fragments Th^−^ (D, 7s^0^ 6d^4^ 5f^1^) and (CO)_8_. The remaining deformation densities of the latter fragmentation scheme are shown in Figure S7 (see the Supporting Information); they look very similar to the deformation densities displayed in Figure 10.

**Figure 11:**
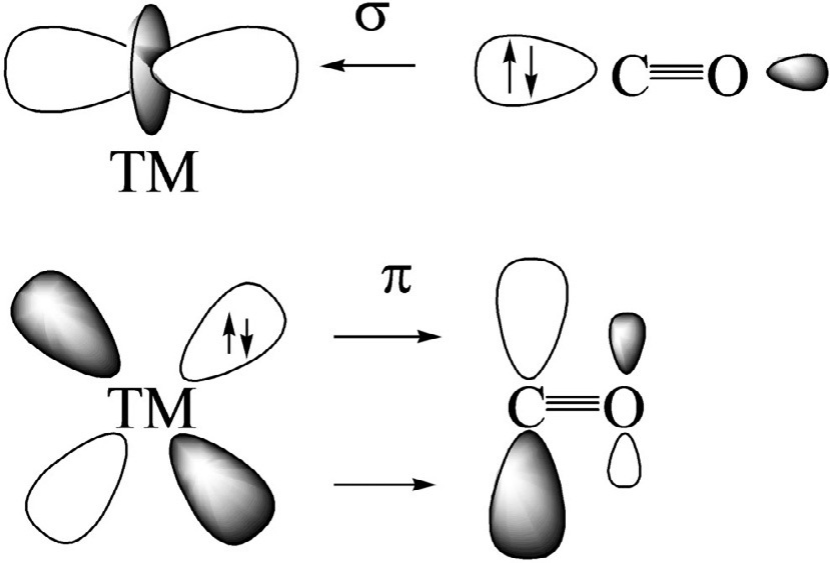
Principal components of the Dewar–Chatt–Duncanson model for transition‐metal carbonyl complexes in terms of TM←CO σ donation and TM→CO π backdonation.

It is illuminating to compare the calculated charge transfer with the calculated and experimentally observed redshift of the C−O vibrational frequencies in the metal complexes (Table 2). It is useful for this purpose to identify the factors that are responsible for the change in the C−O stretching mode. Figure 11 shows the principal components of the Dewar–Chatt–Duncanson (DCD)^38^ model for carbonyl complexes [M−CO], which rationalize the variation of the CO bond in terms of M←CO σ donation and M→CO π backdonation. The latter interaction leads to charge accumulation in the antibonding π* MO of CO, which explains the bond lengthening and the redshift towards lower wavenumbers. The majority of carbonyl complexes exhibits a redshift of the C−O stretching mode. They have, therefore, been termed as normal carbonyl complexes.^39^ Complexes in which the M←CO σ donation dominates the orbital interaction show a blueshift of the stretching mode, and they are called abnormal carbonyl complexes. The reason why M←CO σ donation leads to bond shortening and a blueshift is not directly obvious. It was originally proposed that the σ HOMO of CO is an antibonding orbital,^40^ but inspection of the shape of the orbital did not reveal a node. It was suggested that the blueshift caused by M←CO σ donation is a charge polarization effect rather than a genuine orbital interaction.^41^ Protonation at the carbon or oxygen atom of CO leads to an opposite change of the C−O bond length. The C−O bond in HCO^+^ is shorter and has a blueshift with respect to the free CO, whereas HOC^+^ has a longer bond length and the C−O frequency is redshifted. If the σ HOMO of CO would be an antibonding orbital, it should lead to a shorter C−O bond in HCO^+^ and HOC^+^. The positive charge attached to the carbon atom in HCO^+^ makes the C−O bond less polar and more “N_2_‐like”, which enhances the triple‐bond character, whereas the opposite effect takes in HOC^+^.^41^ This nicely explains the observed changes in the two cations. An important conclusion is that the redshift in normal carbonyl complexes is more sensitive and requires less charge migration owing to M→CO π backdonation than the blueshift in abnormal complexes, which is an indirect effect of the charge polarization. This is important for the present systems.

Table 5 shows the net charge at the metal atoms in [An(CO)_8_]^+/−^, which come from the eigenvalues of the pairwise orbital interactions that are given in Tables 3 and 4. The contributions of the remaining orbitals are neglected. They are mainly due to intrafragment charge polarization and core orbitals. It is interesting to compare the EDA‐NOCV charges with the values from other popular charge partitioning methods, which are also given in Table 5. The net charges of the EDA‐NOCV method suggest that the metal atoms in the anions [An(CO)_8_]^−^ are strong donors, which agrees with the large redshifts of the adducts (Table 2). The thorium atom in [Th(CO)_8_]^−^ donates all of the negative charge to the ligands when Th+(CO)_8_ are used as fragments, whereas the uranium atom in [U(CO)_8_]^−^ even carries a positive charge. The actinide atoms in the [An(CO)_8_]^+^ cations are charge acceptors, but the overall charge transfer An^+^←(CO)_8_ is smaller than in the anions. The effect of the π backdonation in the cations An^+^→(CO)_8_ on the C−O stretching frequencies is stronger than the An^+^←(CO)_8_ σ donation, but the redshifts in the cations [An(CO)_8_]^+^ are much weaker than in the anions [An(CO)_8_]^−^. The calculated net charges of the EDA‐NOCV method agree quite well with the values of the Hirshfeld^42^ partitioning scheme, and in particular, with the recently proposed extended Hirshfeld variant CM5.^43^ A reasonably good agreement of the EDA‐NOCV charges is also found with the Voronoi^44^ results. Surprisingly, different charges are predicted by the popular NBO method.^45^ According to NBO 6.0,^46^ U^−^ is an electron acceptor in [U(CO)_8_]^−^ with a negative charge of −1.33 e. This disagrees with all other values in Table 5, and it is also in conflict with the large redshift in [U(CO)_8_]^−^, which suggests strong U^−^→(CO)_8_ π backdonation.

**Table 5:** Calculated atomic partial charges at An in [An(CO)_8_]^*q*^ (*q*=+1, −1) complexes at the B3LYP‐D3(BJ)/def2‐TZVPPD/Stuttgart RSC ECP level by using various methods.

Complexes	EDA‐NOCV	Hirshfeld	CM5	Voronoi	NBO 6.0	Spin density at the metal
	*q*(An)					
Th(CO)_8_^+^ (*D*_4*h*_, ^2^A_1g_)	0.23	0.25	0.35	0.12	−0.25	0.58
Th(CO)_8_^−^ (*D*_4*h*_, ^2^B_1u_)	0.01^[a]^ (0.22)^[b]^	−0.01	0.13	0.03	−0.79	0.13
U(CO)_8_^+^ (*O_h_*, ^6^A_1g_)	0.61	0.30	0.56	0.17	−0.23	3.80
U(CO)_8_^−^ (*O_h_*, ^4^A_1g_)	0.34	0.07	0.45	0.17	−1.33	2.51

[a] Using Th^−^+(CO)_8_ as fragments. [b] Using Th+(CO)_8_^−^ as fragments.

The NBO method suggests that all metal atoms in the cations and anions [An(CO)_8_]^+/−^ carry a negative partial charge, which is at odds with the results of all other methods. It is also difficult to reconcile the NBO charges with the frequency shifts of the carbonyl ligands. The peculiar NBO charges may be caused by the unbalanced treatment of the valence orbitals of the metals in the sequence of orthogonalization steps in the NBO algorithm. The NBO method makes a preselection of those orbitals, which are considered as genuine atomic valence orbitals for the construction of the molecular orbitals. Only those outermost AOs that are occupied in the electronic ground state of the atom are considered as valence AOs, whereas vacant AOs are named Rydberg AOs. Valence AOs were then favored over Rydberg AOs in the NBO algorithm, which yields MOs that are biased towards the chosen AOs. This can lead to curious and inept results, because atoms may sometimes use AOs for chemical bonding that are vacant in the electronic ground state. Some examples were recently reported by us.^8^, ^17^ We think that the NBO partial charges in Table 5 do not provide a reasonable description of the charge distribution in the actinide ions [An(CO)_8_]^+/−^.

Our experimental results for [U(CO)_8_]^+^ agree quite well with the previous study by Duncan and co‐workers^13^ but the theoretical results are at variance with their study. The previous DFT calculations suggest a *D*_4*d*_ equilibrium geometry and a redshift of the C−O stretching mode by 80 cm^−1^. Our calculations give an *O_h_* structure and a smaller redshift of 63 cm^−1^, which agrees better with the experimental value of −56 cm^−1^ in our work and the observed data of −63 cm^−1^ by Duncan and co‐workers.^13^ We think that the difference may come from the choice of a different electron configuration for the sextet state of [U(CO)_8_]^+^ in their work. The authors carried out CASSCF calculations by stating that the five unpaired electrons “reside primarily in the 5f and 7s orbitals.” ^[13]^ This is at odds with our results, which give the electron configuration of 7s^0^ 7p^0^ 6d^2^ 5f^3^ at the uranium atom, in which two unpaired electrons are in 6d AOs. The EDA‐NOCV analysis suggests that the energy contribution through donation from the 6d electrons in the occupied e_g_ AO and the backdonation into the vacant 6d AOs (t_2g_) are much larger than the contribution of the occupied and vacant 5f AOs (Table 3). We found that the energetically lowest lying sextet state of [U(CO)_8_]^+^ possesses *O_h_* symmetry. We searched for a *D*_4*d*_ structure with an electron sextet state of [U(CO)_8_]^+^ and found an energy minimum structure with the electron configuration of 7s^0^ 7p^0^ 6d^1^ 5f^4^, which is 24.8 kcal mol^−1^ higher in energy than the *O_h_* structure with the electron configuration of 7s^0^ 7p^0^ 6d^2^ 5f^3^ (see the Supporting Information, Figure S4). The *D*_4*d*_ structure exhibits frequency shifts of the C−O stretching mode of 80 and 85 cm^−1^ (see the Supporting Information, Table S2), which agrees with the reported value of 80 cm^−1^ by Duncan and co‐workers.^13^, ^47^ As the calculated redshift of the *O_h_* structure is in better agreement with the experimental values and clearly lower in energy than the *D*_4*d*_ structure, we think that the computed *D*_4*d*_ structure of [U(CO)_8_]^+^ in the previous work refers to an excited sextet state of the molecule.

The choice of the electron configuration of the uranium atom has a strong effect on the analysis of the metal–CO bonds in [U(CO)_8_]^+^. Ricks et al. interpreted the redshift in terms of U^+^(5f)→CO π backdonation by saying that “apparently, back‐donation from f orbitals is also quite efficient, even though the spatial overlap is quite different from that of d orbitals.”^13^ Our results support this statement, but the main component of U^+^→CO π backdonation comes from the two electrons in the 6d AOs. This is in agreement with previous theoretical studies on multiple bonds in terminal actinide complexes L_*n*_An≡E (An=Pa, U, Np, Pu; E=N, P, As, Sb, Bi), which suggest that the 6d orbitals of the actinides are more important for chemical bonding than the 5f orbitals.^48^ Further theoretical studies about different classes of actinide compounds are warranted to provide a definite statement about the relevance of the 6d and 5f AOs of the metals for chemical bonding.

A final word shall be devoted to the 32‐electron rule,^2^ which is obviously not valid for the studied systems [An(CO)_8_]^+/−^. In the work by Duncan and co‐workers, it was suggested that “the d orbitals in actinides are usually less important in bonding, and therefore, stable actinide systems often have 22 electrons [e.g., U(C_8_H_8_)_2_].”^13^ Our results evidently do not support a 22‐electron rule for actinides based on dominantly 5f AOs. It seems that steric and electronic effects determine the stability of actinide complexes in concert. We shall analyze the nature of the bonding in U(C_8_H_8_)_2_ and other stable actinide complexes in future studies.

## Conclusion

The octacarbonyl cation and anion complexes of actinide metals [An(CO)_8_]^+/−^ (An=Th, U) were prepared in the gas phase, and they were studied by mass‐selected infrared photodissociation spectroscopy. Both the octacarbonyl cations and the anions were characterized as saturated coordinated complexes. Quantum chemical calculations that used density functional theory show that the [Th(CO)_8_]^+^ and [Th(CO)_8_]^−^ complexes have a distorted octahedral (*D*_4*h*_) equilibrium geometry and a doublet electronic ground state. Both the [U(CO)_8_]^+^ cation and the [U(CO)_8_]^−^ anion exhibit cubic structures (*O_h_*) with a ^6^A_1g_ ground state for the cation and a ^4^A_1g_ ground state for the anion. The neutral species [Th(CO)_8_] (*O_h_*; ^1^A_1g_) and [U(CO)_8_] (*D*_4*h*_; ^5^B_1u_) have also been calculated. Analysis of their electronic structures with the help on an energy decomposition method revealed that, along with the dominant 6d valence orbitals, there is significant 5f orbital participation in both the [An]←CO σ donation and [An]→CO π back donation interactions in the cations and anions, for which the electronic reference state of An has both occupied and vacant 5f AOs. The trend of the valence orbital contribution to the metal–CO bonds has the order of 6d≫5f>7s≈7p, with the 5f orbitals of uranium being more important that the 5f orbitals of thorium.

## Conflict of interest

The authors declare no conflict of interest.

## Supporting information

As a service to our authors and readers, this journal provides supporting information supplied by the authors. Such materials are peer reviewed and may be re‐organized for online delivery, but are not copy‐edited or typeset. Technical support issues arising from supporting information (other than missing files) should be addressed to the authors.

Supplementary
