# Supplementary material for: Octacarbonyl Ion Complexes of Actinides [An(CO)8]+/− (An=Th, U) and the Role of f Orbitals in Metal–Ligand Bonding
Source: Chemistry. 2019 Aug 23;25(50):11772–84. doi: 10.1002/chem.201902625 (PMC6772027; doi:10.1002/chem.201902625)
Supplement: Supplementary file 1 — Supplementary [file CHEM-25-11772-s001.pdf]

CHEMISTRY
A European Journal

Supporting Information

Octacarbonyl Ion Complexes of Actinides $[An(CO)_8]^{+/-}$ (An = Th, U) and the Role of f Orbitals in Metal–Ligand Bonding

Chaoxian Chi+,[a] Sudip Pan+,[b] Jiaye Jin,[c] Luyan Meng,[a] Mingbiao Luo,[a] Lili Zhao,[b] Mingfei Zhou,*[c] and Gernot Frenking*[b, d]

chem_201902625_sm_miscellaneous_information.pdf

Octacarbonyl Ion Complexes of Actinides $[An(CO)_8]^{+/-}$ (An = Th, U) and the Role of f Orbitals in Metal-Ligand Bonding

Supporting Information

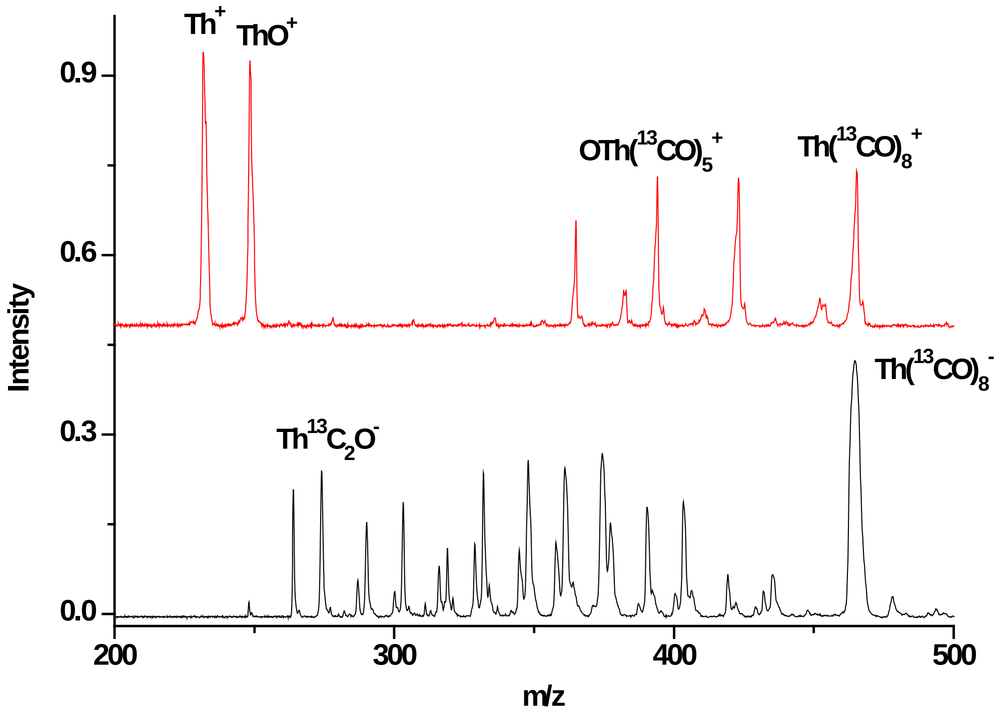

Figure S1. Mass spectra of thorium carbonyl cation and anion complexes using ^{13}CO.

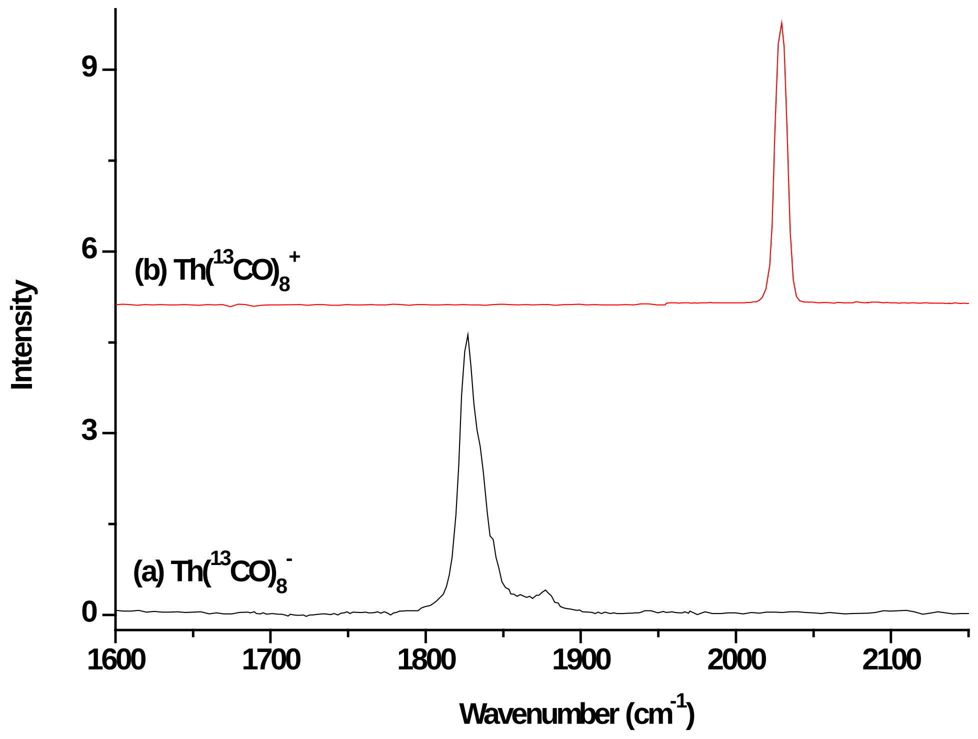

Figure S2. Infrared photodissociation spectra of (a) $Th(^{13}CO)_8^-$, and (b) $Th(^{13}CO)_8^+$.

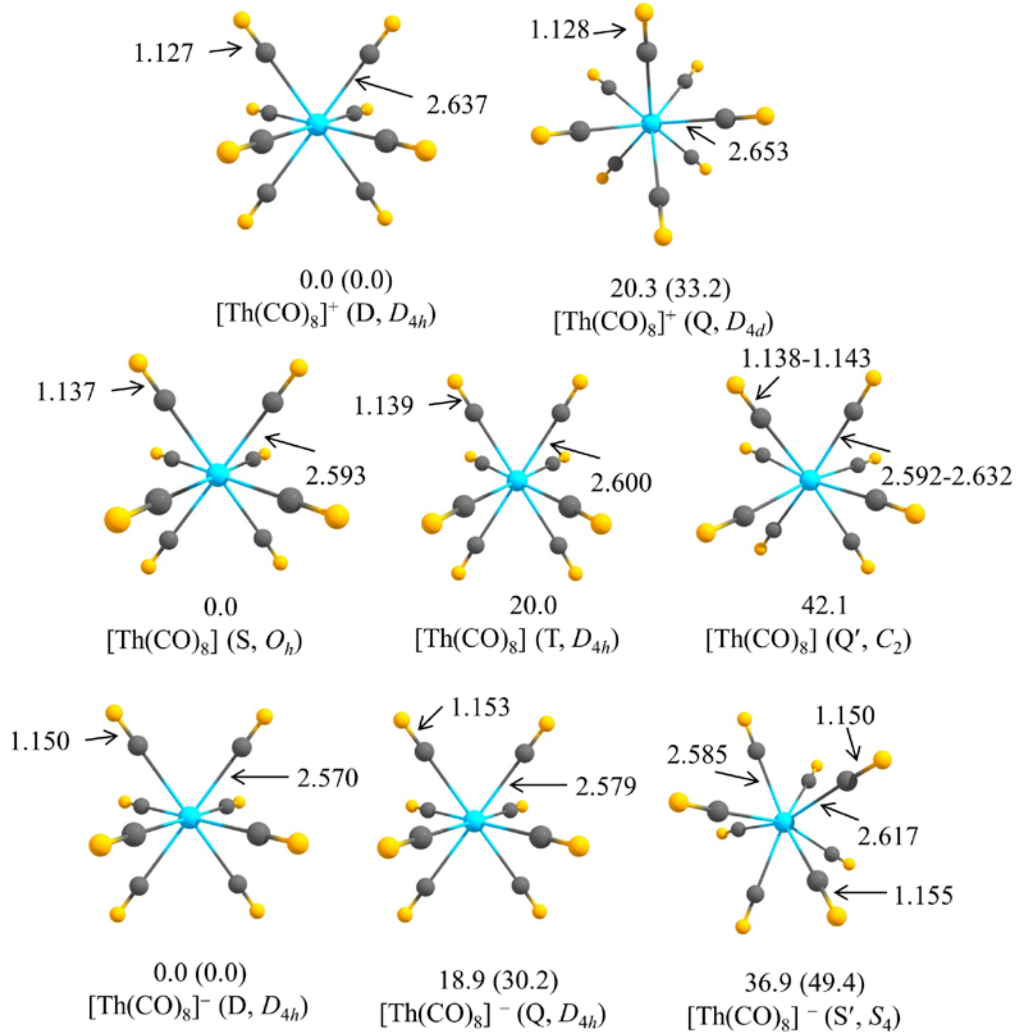

Figure S3. Calculated equilibrium geometries of $[Th(CO)_8]^q$ (q = +1, 0, -1) complexes with different spin states at the B3LYP-D3(BJ)/def2-TZVPPD/Stuttgart RSC ECP level. Relative energies are in kcal/mol. The relative energies in parentheses are at the CCSD(T)/def2-SVP/Stuttgart RSC ECP//B3LYP-D3(BJ)/def2-TZVPPD/Stuttgart RSC ECP level.

Q = Quartet; Q′ = Quintet; S′ = Sextet

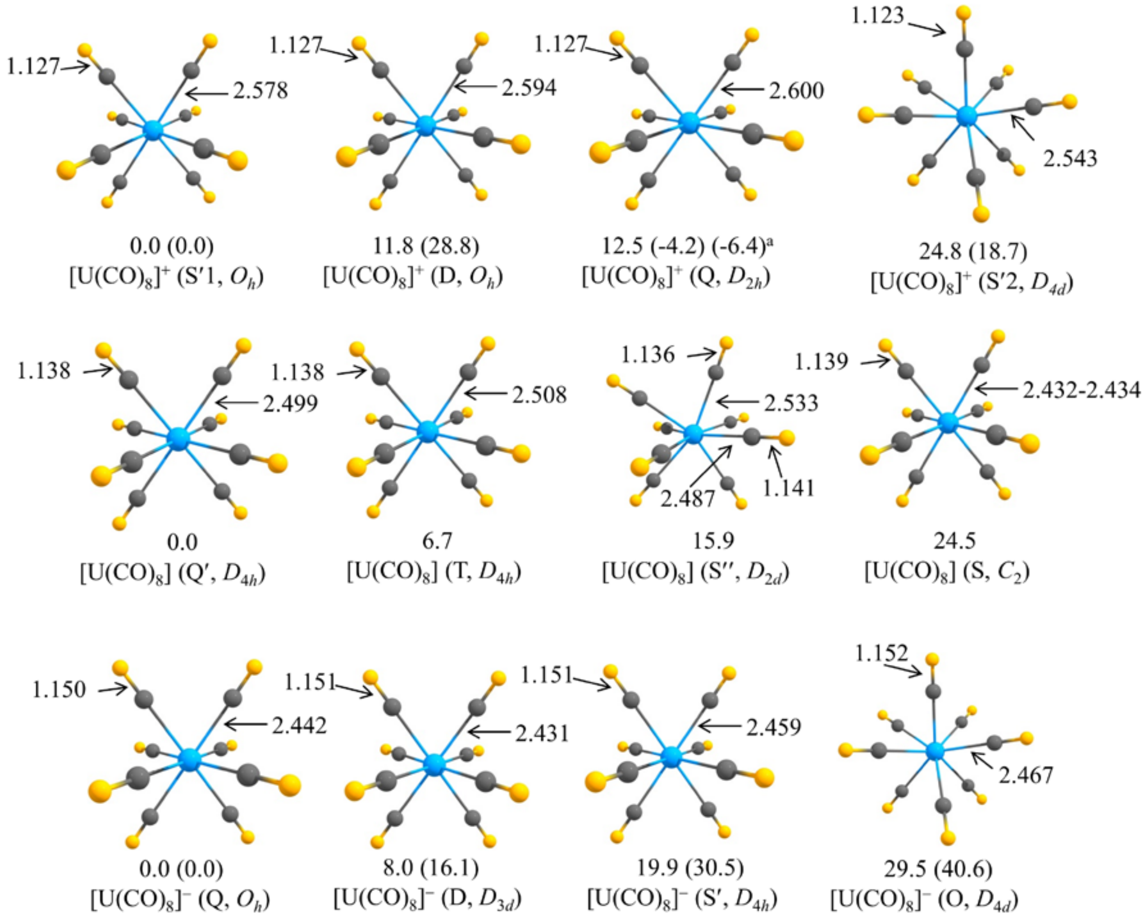

Figure S4. Calculated equilibrium geometries of $[U(CO)_8]^q$(q = +1, 0, -1) complexes with different spin states at the B3LYP-D3(BJ)/def2-TZVPPD/Stuttgart RSC ECP level. Relative energies are in kcal/mol. The relative energies in parentheses are at the CCSD(T)/def2-SVP/Stuttgart RSC ECP//B3LYP-D3(BJ)/def2-TZVPPD/Stuttgart RSC ECP level. [a]The value is at the CCSD(T)/def2-TZVP//B3LYP-D3(BJ)/def2-TZVPPD/Stuttgart RSC ECP level.

Q = Quartet; Q′ = Quintet; S′ = Sextet; S′′ = Septet; O = Octet
S′1 = Sextet with electron configuration ($7s^07p^06d^25f^3$).
S′2 = Sextet with electron configuration ($7s^07p^06d^15f^4$).

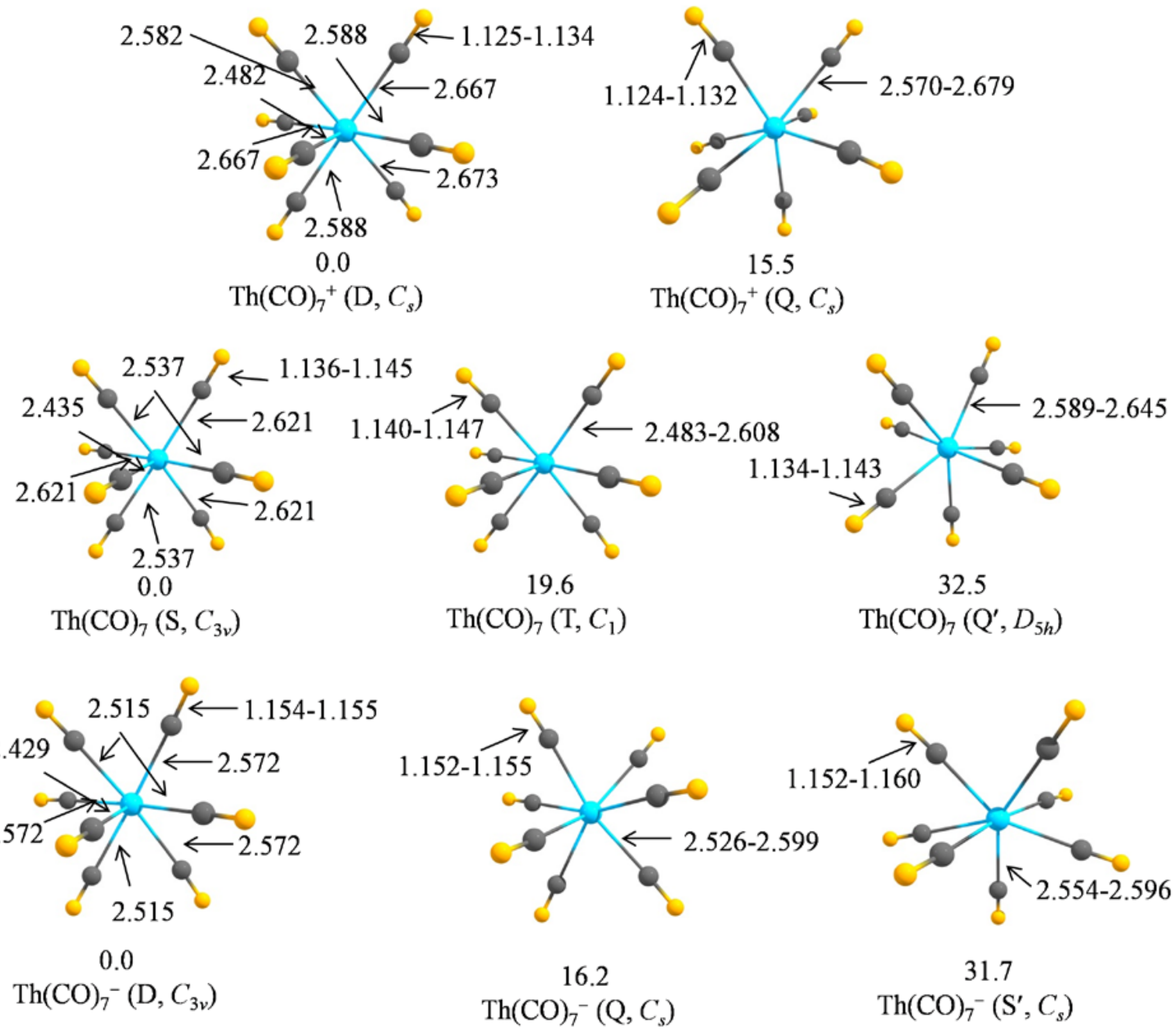

Figure S5. Calculated equilibrium geometries of $[Th(CO)_7]^q$ (q = +1, 0, -1) complexes with different spin states at the B3LYP-D3(BJ)/def2-TZVPPD/Stuttgart RSC ECP level. Relative energies are in kcal/mol. Bond distances are in Å.

Q = Quartet; Q′ = Quintet; S′ = Sextet

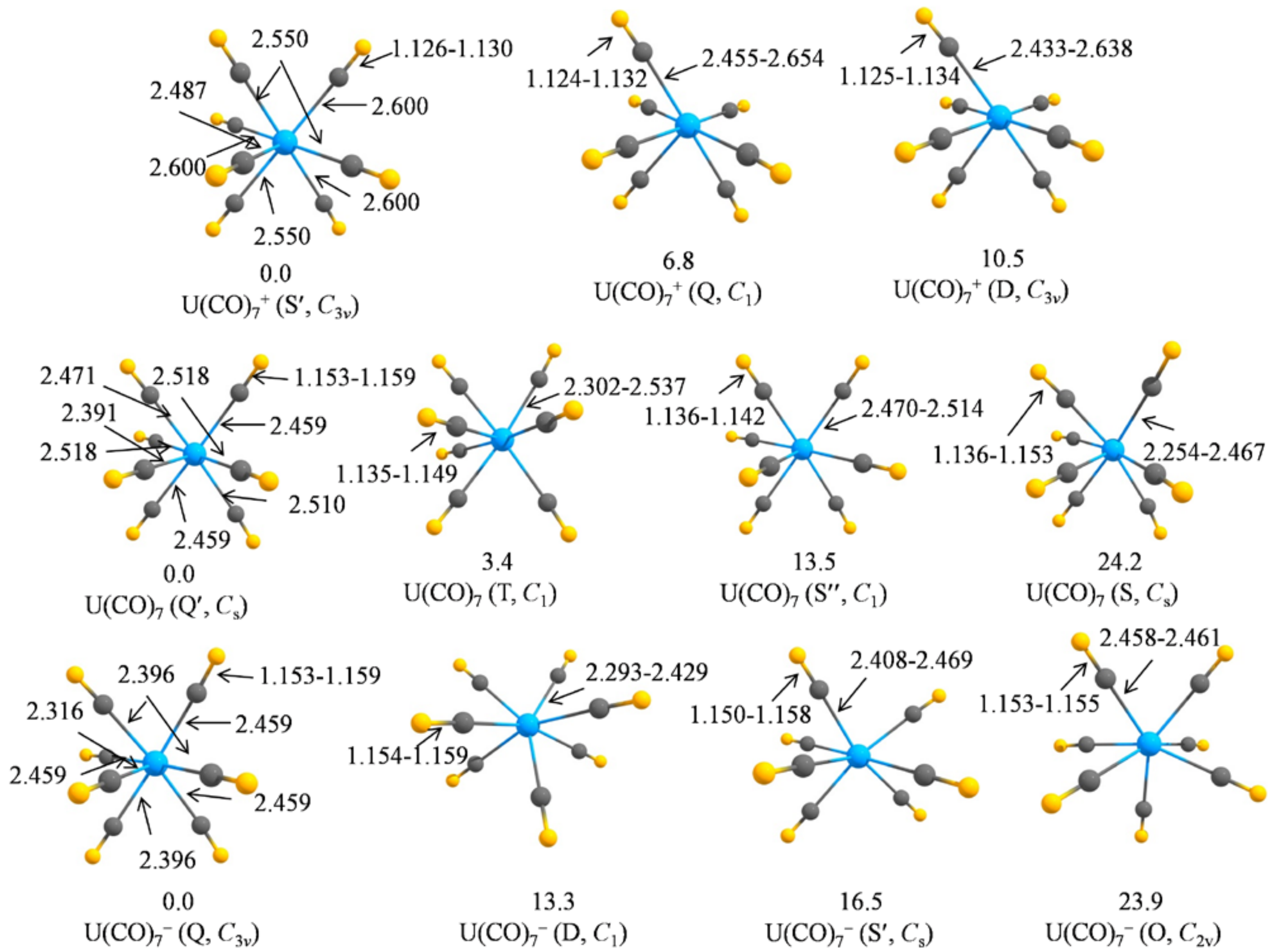

Figure S6. Calculated equilibrium geometries of $[U(CO)_7]^q$ (q = +1, 0, -1) complexes with different spin states at the B3LYP-D3(BJ)/def2-TZVPPD/Stuttgart RSC ECP level. Relative energies are in kcal/mol. Bond distances are in Å.

Q = Quartet; Q′ = Quintet; S′ = Sextet; S′′ = Septet; O = Octet

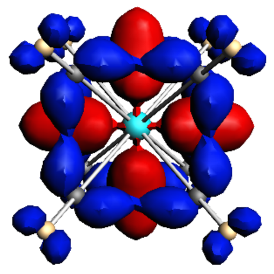<br>**$\Delta\rho_{(1)}$ ($2b_{2g}$)**<br>**$\Delta E_{orb(1)}$ = -125.7**<br>**$\lvert\nu_1\rvert$ = 1.30**	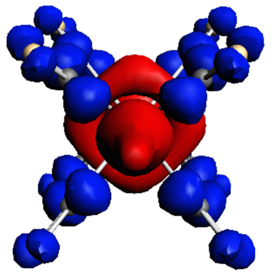<br>**$\Delta\rho_{(2)}$ ($2a_{1g}$)**<br>**$\Delta E_{orb(2)}$ = -129.0**<br>**$\lvert\nu_2\rvert$ = 1.20**	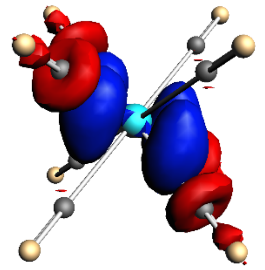<br>**$\Delta\rho_{(3a)}$ (e_g)**<br>**$\Delta E_{orb(3a)}$ = -20.8 (2x)**<br>**$\lvert\nu_{3b}\rvert$ = 0.40**	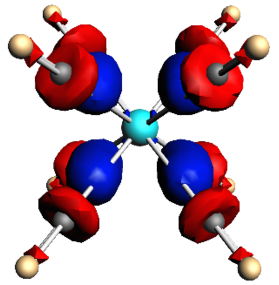<br>**$\Delta\rho_{(4)}$ (b_{1g})**<br>**$\Delta E_{orb(4)}$ = -19.2**<br>**$\lvert\nu_4\rvert$ = 0.41**
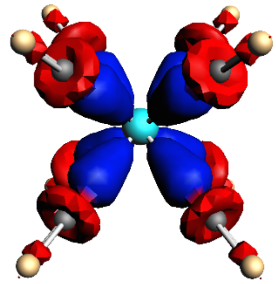<br>**$\Delta\rho_{(5)}$ (b_{2u})**<br>**$\Delta E_{orb(5)}$ = -13.4**<br>**$\lvert\nu_5\rvert$ = 0.38**	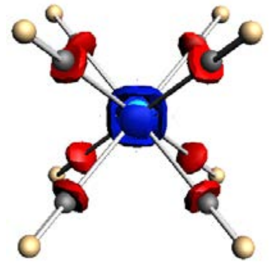<br>**$\Delta\rho_{(6)}$ (a_{1g})**<br>**$\Delta E_{orb(6)}$ = -4.3**<br>**$\lvert\nu_6\rvert$ = 0.19**	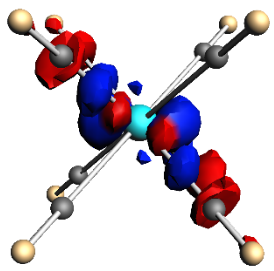<br>**$\Delta\rho_{(7a)}$ (e_u)**<br>**$\Delta E_{orb(7a)}$ = -3.2 (2x)**<br>**$\lvert\nu_{7a}\rvert$ =0.20**	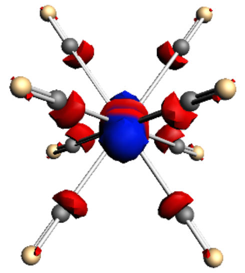<br>**$\Delta\rho_{(8)}$ (a_{2u})**<br>**$\Delta E_{orb(8)}$ = -1.5**<br>**$\lvert\nu_8\rvert$ = 0.14**

Figure S7. Shape of the deformation densities $\Delta\rho_{(1)\text{-}(8)}$, which are associated with the orbital interactions $\Delta E_{orb(1)\text{-}(8)}$ in $[Th(CO)_8]^-$ complex for fragments, Th^- (D, $7s^0 6d^4 5f^1$) and $(CO)_8$(S)and eigenvalues $\lvert\nu_n\rvert$ of the charge flow. The isosurface values are 0.002 for $\Delta\rho_{(1)}$ and $\Delta\rho_{(2)}$, and 0.001 for $\Delta\rho_{(3)\text{-}(8)}$. The color code of the charge flow is red → blue. Energies are in kcal/mol.

Table S1. The T_1 diagnostic value of $[An(CO)_8]^q$ (q = +1, 0, -1) complexes at the CCSD(T)/def2-SVP//B3LYP-D3(BJ)/def2-TZVPPD/Stuttgart RSC ECP level.

Complexes	T_1
$[Th(CO)_8]^+$ (D_{4h}, $^2A_{1g}$)	0.0204
$[Th(CO)_8]$ (O_h, $^1A_{1g}$)	0.0208
$[Th(CO)_8]^-$ (D_{4h}, $^2B_{1u}$)	0.0217
$[U(CO)_8]^+$ (O_h, $^6A_{1g}$)	0.0259
$[U(CO)_8]^+$ (D_{2h}, $^4B_{2u}$)	0.0223
$[U(CO)_8]$ (D_{4h}, $^5B_{1u}$)	0.0304
$[U(CO)_8]^-$ (O_h, $^4A_{1g}$)	0.0327

Table S2. Calculated[a] (B3LYP-D3(BJ)/def2-TZVPPD/ECP) and experimental C-O stretching wavenumbers (cm^{-1}) for the $[U(CO)_8]^+$ (D_{2h}, $^4B_{2u}$) and $[U(CO)_8]^+$ (D_{4d}, $^6A_1$) complexes. Calculated IR intensities in km mol^{-1} are given in parentheses.

	Calcd.			Exptl.	
	^{12}CO	$\Delta(CO)^b$		^{12}CO	$\Delta(CO)^b$
$U(CO)_8^+$ (D_{2h}, $^4B_{2u}$)	2142 (0, a_g)				
	2083 (0, b_{2g})				
	2082 (0, a_u)				
	2080 (1918, b_{2u})	-63		2087	-56
	2079 (0, b_{1g})				
	2072 (2527, b_{1u})	-71			
	2070 (2295, b_{3u})	-73			
	2068 (0, b_{3g})				
$[U(CO)_8]^+$ (D_{4d}, $^6A_1$)	2139 (0, a_1)				
	2068 (0, e_2)				
	2064 (0, e_2)				
	2063 (1814, b_2)	-80			
	2058 (2900, e_2)	-85			

[a]Scaled by 0.968 using the ratio of calculated (2213 cm^{-1}) and experimental (2143 cm^{-1}) wavenumbers of free CO.
[b]Frequency shift with regard to free CO.

Table S3. The Cartesian coordinates and the total energies at the B3LYP-D3(BJ)/def2-TZVPPD/Stuttgart RSC 1997 ECP level.

$[Th(CO)_8]^+$ (D_{4h}, D)

E = -1314.5616634 au

C	0.000000000	2.190400000	1.467925000
O	0.000000000	3.132330000	2.086747000
C	2.190400000	0.000000000	1.467925000
C	0.000000000	-2.190400000	1.467925000
C	0.000000000	2.190400000	-1.467925000
C	-2.190400000	0.000000000	-1.467925000
C	2.190400000	0.000000000	-1.467925000
C	0.000000000	-2.190400000	-1.467925000
O	3.132330000	0.000000000	2.086747000
O	0.000000000	-3.132330000	2.086747000
O	0.000000000	3.132330000	-2.086747000
O	-3.132330000	0.000000000	-2.086747000
O	3.132330000	0.000000000	-2.086747000
O	0.000000000	-3.132330000	-2.086747000
C	-2.190400000	0.000000000	1.467925000
O	-3.132330000	0.000000000	2.086747000
Th	0.000000000	0.000000000	0.000000000

$[Th(CO)_8]$ (O_h, S)

E = -1314.8084453 au

C	1.496794000	1.496794000	1.496794000
O	2.153143000	2.153143000	2.153143000
C	-1.496794000	1.496794000	1.496794000
C	-1.496794000	1.496794000	-1.496794000
C	1.496794000	-1.496794000	1.496794000
C	1.496794000	-1.496794000	-1.496794000
C	-1.496794000	-1.496794000	1.496794000
C	-1.496794000	-1.496794000	-1.496794000
O	-2.153143000	2.153143000	2.153143000
O	-2.153143000	2.153143000	-2.153143000
O	2.153143000	-2.153143000	2.153143000
O	2.153143000	-2.153143000	-2.153143000
O	-2.153143000	-2.153143000	2.153143000
O	-2.153143000	-2.153143000	-2.153143000
C	1.496794000	1.496794000	-1.496794000
O	2.153143000	2.153143000	-2.153143000
Th	0.000000000	0.000000000	0.000000000

$[Th(CO)_8]^-$ (D_{4h}, D)
E = -1314.8721094 au

C	0.000000000	1.953331000	1.670517000
O	0.000000000	2.803700000	2.445165000
C	0.000000000	1.953331000	-1.670517000
C	1.953331000	0.000000000	-1.670517000
C	-1.953331000	0.000000000	1.670517000
C	0.000000000	-1.953331000	1.670517000
C	-1.953331000	0.000000000	-1.670517000
C	0.000000000	-1.953331000	-1.670517000
O	0.000000000	2.803700000	-2.445165000
O	2.803700000	0.000000000	-2.445165000
O	-2.803700000	0.000000000	2.445165000
O	0.000000000	-2.803700000	2.445165000
O	-2.803700000	0.000000000	-2.445165000
O	0.000000000	-2.803700000	-2.445165000
C	1.953331000	0.000000000	1.670517000
O	2.803700000	0.000000000	2.445165000
Th	0.000000000	0.000000000	0.000000000

$[U(CO)_8]^+$ (O_h, Sextet)
E = -1383.8029284 au

C	1.488345000	1.488345000	1.488345000
O	2.139022000	2.139022000	2.139022000
C	-1.488345000	1.488345000	1.488345000
C	-1.488345000	1.488345000	-1.488345000
C	1.488345000	-1.488345000	1.488345000
C	1.488345000	-1.488345000	-1.488345000
C	-1.488345000	-1.488345000	1.488345000
C	-1.488345000	-1.488345000	-1.488345000
O	-2.139022000	2.139022000	2.139022000
O	-2.139022000	2.139022000	-2.139022000
O	2.139022000	-2.139022000	2.139022000
O	2.139022000	-2.139022000	-2.139022000
O	-2.139022000	-2.139022000	2.139022000
O	-2.139022000	-2.139022000	-2.139022000
C	1.488345000	1.488345000	-1.488345000
O	2.139022000	2.139022000	-2.139022000
U	0.000000000	0.000000000	0.000000000

$[U(CO)_8]$ (D_{4h}, Quintet)

E = -1384.046101 au

C 0.00000000 2.01065300 1.48397900
O 0.00000000 2.91809300 2.17054700
C 2.01065300 0.00000000 1.48397900
C 0.00000000 -2.01065300 1.48397900
C 0.00000000 2.01065300 -1.48397900
C -2.01065300 0.00000000 -1.48397900
C 2.01065300 0.00000000 -1.48397900
C 0.00000000 -2.01065300 -1.48397900
O 2.91809300 0.00000000 2.17054700
O 0.00000000 -2.91809300 2.17054700
O 0.00000000 2.91809300 -2.17054700
O -2.91809300 0.00000000 -2.17054700
O 2.91809300 0.00000000 -2.17054700
O 0.00000000 -2.91809300 -2.17054700
C -2.01065300 0.00000000 1.48397900
O -2.91809300 0.00000000 2.17054700
U 0.00000000 0.00000000 0.00000000

$[U(CO)_8]^-$ (O_h, Quartet)
E = -1384.1473726 au

C	1.410165000	1.410165000	1.410165000
O	2.074373000	2.074373000	2.074373000
C	-1.410165000	1.410165000	1.410165000
C	-1.410165000	1.410165000	-1.410165000
C	1.410165000	-1.410165000	1.410165000
C	1.410165000	-1.410165000	-1.410165000
C	-1.410165000	-1.410165000	1.410165000
C	-1.410165000	-1.410165000	-1.410165000
O	-2.074373000	2.074373000	2.074373000
O	-2.074373000	2.074373000	-2.074373000
O	2.074373000	-2.074373000	2.074373000
O	2.074373000	-2.074373000	-2.074373000
O	-2.074373000	-2.074373000	2.074373000
O	-2.074373000	-2.074373000	-2.074373000
C	1.410165000	1.410165000	-1.410165000
O	2.074373000	2.074373000	-2.074373000
U	0.000000000	0.000000000	0.000000000

$Th(CO)_7^+$ (C_s, D)
E = -1201.166416 au

C	1.044725000	-0.840483000	2.114861000
C	2.642205000	0.886366000	0.000000000
C	-0.990888000	1.244143000	-2.255444000

C	-0.990888000	1.244143000	2.255444000
C	1.044725000	-0.840483000	-2.114861000
C	-2.465930000	-0.347897000	-0.000000000
O	-1.440297000	1.583774000	-3.229687000
O	1.466835000	-1.348253000	-3.030606000
O	1.466835000	-1.348253000	3.030606000
O	-1.440297000	1.583774000	3.229687000
O	-3.556054000	-0.642713000	-0.000000000
O	3.745646000	1.106067000	0.000000000
C	-0.416236000	-2.175071000	-0.000000000
O	-0.623029000	-3.289704000	-0.000000000
Th	0.042629000	0.264646000	0.000000000

$Th(CO)_7$ (C_{3v}, S)

E = -1201.412052 au

C 2.18090500 -1.25914600 1.05173300
C 0.00000000 -2.33382500 -0.66776700
C 0.00000000 2.51829300 1.05173300
C -2.02115200 1.16691200 -0.66776700
C 2.02115200 1.16691200 -0.66776700
C 0.00000000 0.00000000 -2.10840000
O 3.14836500 -1.81770900 1.25676000
O 0.00000000 -3.36176500 -1.16103100
O 0.00000000 3.63541900 1.25676000
O -2.91137400 1.68088200 -1.16103100
O 2.91137400 1.68088200 -1.16103100
O 0.00000000 0.00000000 -3.25369900
C -2.18090500 -1.25914600 1.05173300
O -3.14836500 -1.81770900 1.25676000
Th 0.00000000 0.00000000 0.32614000

$Th(CO)_7^-$ (C_{3v}, D)

E = -1201.474126 au

C	-0.000000000	2.371829000	-0.470912000
C	-2.195014000	1.267292000	0.804232000
C	-0.000000000	-2.534584000	0.804232000
C	2.195014000	1.267292000	0.804232000
C	-2.054065000	-1.185915000	-0.470912000
C	2.054065000	-1.185915000	-0.470912000
O	0.000000000	-3.687580000	0.863033000
O	-2.984472000	-1.723086000	-0.894218000
O	0.000000000	3.446171000	-0.894218000
O	3.193538000	1.843790000	0.863033000
O	2.984472000	-1.723086000	-0.894218000

O	-3.193538000	1.843790000	0.863033000
C	0.000000000	0.000000000	-2.063463000
O	0.000000000	0.000000000	-3.217709000
Th	-0.000000000	-0.000000000	0.365302000

$U(CO)_7^+$ (C_{3v}, Sextet)
E = -1270.4088630 au

C	0.000000000	-2.390345000	-0.651506000
C	2.112818000	-1.219836000	1.136002000
C	0.000000000	2.439672000	1.136002000
C	-2.112818000	-1.219836000	1.136002000
C	2.070099000	1.195173000	-0.651506000
C	-2.070099000	1.195173000	-0.651506000
O	-0.000000000	3.513594000	1.474498000
O	2.981366000	1.721293000	-1.059158000
O	-0.000000000	-3.442585000	-1.059158000
O	-3.042861000	-1.756797000	1.474498000
O	-2.981366000	1.721293000	-1.059158000
O	3.042861000	-1.756797000	1.474498000
C	-0.000000000	0.000000000	-2.249270000
O	-0.000000000	0.000000000	-3.379002000
U	0.000000000	-0.000000000	0.237395000

$U(CO)_7$ (C_s, Quintet)
E = -1270.6487696 au

C	0.464440000	-1.422108000	2.144176000
O	0.464440000	-1.978726000	3.136727000
C	-0.140055000	1.278901000	1.982668000
C	1.927771000	1.772152000	0.000000000
C	-1.472566000	-1.864081000	0.000000000
C	0.464440000	-1.422108000	-2.144176000
C	-1.960059000	0.735790000	0.000000000
C	-0.140055000	1.278901000	-1.982668000
O	-0.366591000	1.927670000	2.893866000
O	2.628286000	2.669169000	0.000000000
O	-2.302429000	-2.646171000	0.000000000
O	0.464440000	-1.978726000	-3.136727000
O	-3.026785000	1.147301000	0.000000000
O	-0.366591000	1.927670000	-2.893866000
U	0.273678000	-0.116197000	0.000000000

$U(CO)_7^-$ (C_{3v}, Quartet)
E = -1270.7430847 au

C	0.000000000	2.196836000	-0.615058000
C	-2.064885000	1.192162000	0.943745000
C	-0.000000000	-2.384324000	0.943745000
C	2.064885000	1.192162000	0.943745000
C	-1.902516000	-1.098418000	-0.615058000
C	1.902516000	-1.098418000	-0.615058000
O	-0.000000000	-3.525137000	1.110908000
O	-2.798493000	-1.615711000	-1.129299000
O	0.000000000	3.231421000	-1.129299000
O	3.052858000	1.762569000	1.110908000
O	2.798493000	-1.615711000	-1.129299000
O	-3.052858000	1.762569000	1.110908000
C	0.000000000	0.000000000	-1.973831000
O	0.000000000	0.000000000	-3.132616000
U	-0.000000000	-0.000000000	0.341778000
